# Surface Reconstruction in Quasi‐2D Perovskite Films Treated with Cesium Halide Nanocrystals: Halide Exchange or Phase Transformation

**DOI:** 10.1002/smsc.202500163

**Published:** 2025-07-08

**Authors:** Dong Il Son, Seonhong Min, Sohyeon An, Dongryeol Lee, Se Hyun Lee, Donghan Kim, Myoung Hoon Song, Jin Young Kim, Sungwook Park, Junsang Cho, Jongnam Park

**Affiliations:** ^1^ Graduate School of Semiconductor Materials and Device Engineering Ulsan National Institute of Science and Technology (UNIST) Ulsan 44919 Republic of Korea; ^2^ School of Chemistry and Energy Sungshin Women's University Seoul 01133 Republic of Korea; ^3^ School of Energy and Chemical Engineering Ulsan National Institute of Science and Technology (UNIST) Ulsan 44919 Republic of Korea; ^4^ Department of Materials Science and Engineering Ulsan National Institute of Science and Technology (UNIST) Ulsan 44919 Republic of Korea; ^5^ Graduate School of Carbon Neutrality Ulsan National Institute of Science and Technology (UNIST) Ulsan 44919 Republic of Korea; ^6^ Research and Development Center UniQDot Ulsan 44919 Republic of Korea; ^7^ Department of Biomedical Engineering Ulsan National Institute of Science and Technology (UNIST) Ulsan 44919 Republic of Korea

**Keywords:** cesium halide treatments, halide exchanges, in situ spectroscopic analyses, phase transformations, quasi‐2D perovskite films

## Abstract

The formation of heterostructure interfaces from quantum dots (or nanocrystals) and lower‐dimensional (2D or quasi‐2D) materials enables interfacial and optoelectronic property tuning. However, this strategy has not been sufficiently characterized, for example, the application of cesium halide nanocrystals to quasi‐2D perovskite structures is underexplored, and the mechanisms of the resulting structural modifications and specific nanocrystal roles are not fully understood. Herein, the effects of postsynthetically surface‐modifying quasi‐2D perovskite films with CsX (*X* = Cl, Br, I) nanocrystals are examined to bridge this gap. The purposeful choice of *X* enables the selective induction of halide exchange or a structural phase transformation at the nanocrystal–perovskite interface, which leads to optical bandgap and luminescence property modulation over a wide range of the visible spectrum (450–620 nm). Results of in situ spectroscopic analyses and temperature‐dependent kinetic studies reveal that the activation energy for the halide exchange (24–29 kJ mol^−1^) is lower than that for the structural phase transformation to 0D Cs_4_PbX_6_ nanocrystals (39 kJ mol^−1^), indicating the kinetic favorability of the former process. The potential of the developed strategy is showcased through the fabrication of efficient color‐tunable light‐emitting diodes with quasi‐2D perovskite films surface modified with CsX as active emission layers.

## Graphical abstract

Surface modification of quasi‐2D perovskite films with CsX (*X* = Cl, Br, I) nanocrystals enables tunable photoluminescence across the visible spectrum. Selective halide exchange or structural phase transformation occurs at the interface, governed by kinetic differences. This nanoscale strategy reveals interfacial reaction pathways and supports the fabrication of spectrally tunable light‐emitting diodes using engineered perovskite nanostructures.

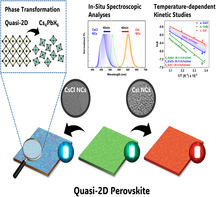

## 1 Introduction

Metal halide perovskites, which have the generic formula ABX_3_ (A^+^ = CH_3_NH_3_^+^ (MA), Cs^+^, HC(NH_2_)_2_^+^ (FA); B^2+^ = Pb^2+^, Sn^2+^; X^−^ = Cl^−^, Br^−^, I^−^), have revolutionized optoelectronics, exhibiting remarkable performance in light‐emitting diodes (LEDs), photodetectors, solar cells, X‐ray scintillators, and lasers.^[^ ^1^, ^2^, ^3^, ^4^, ^5^, ^6^, ^7^, ^8^, ^9^ ^]^ The advantageous properties of these compounds, including high absorption coefficients (≈10^6^ M^−1^ cm^−1^),^[^ ^10^ ^]^ narrow‐bandgap emission, and high photoluminescence quantum yield (PLQY, ≈100%), arise from their defect‐tolerant electronic structures.^[^ ^1^, ^11^, ^12^, ^13^, ^14^ ^]^

Despite this defect tolerance, the presence of vacancies, dangling bonds, and uncoordinated atoms can lead to nonradiative recombination at defect sites and, hence, perovskite degradation.^[^ ^15^, ^16^, ^17^ ^]^ The passivation of perovskite defect states through the use of ligands, additives, and surface treatments can substantially enhance the PLQY, stability, and device performance.^[^ ^18^, ^19^, ^20^ ^]^ Therefore, understanding and controlling the surface chemistry of perovskites are crucial for their further development and application in optoelectronic devices.

Quasi‐2D or 2D/3D perovskites are promising alternatives to conventional 3D perovskites, showing exceptional stabilities and broadly tunable optoelectronic properties.^[^ ^7^, ^21^, ^22^, ^23^, ^24^, ^25^, ^26^, ^27^, ^28^, ^29^ ^]^ The broad structural variability of inorganic and organic components provides a rich design space and numerous degrees of freedom for optoelectronic property (e.g., bandgap, exciton binding energy, and PLQY) tuning. In particular, the tuning of the number of inorganic layers (*n*) within 2D structures enables the facile modulation of the bandgap, which can be further engineered by adjusting the halide composition.^[^ ^30^, ^31^, ^32^, ^33^ ^]^ The adjustment of *n* is achieved by altering the type and ratio of organic spacers, such as phenethylammonium (PEA), butylammonium, or other long‐chain alkylammonium cations,^[^ ^7^ ^]^ which act as barriers between the inorganic layers and influence quantum and dielectric confinement effects.^[^ ^21^ ^]^ Förster‐type resonance energy transfer between adjacent layers with different *n* values results in the cascading energy transfer of excitons toward the lowest‐bandgap phase. The increase in carrier density, facilitated by the ideal energy transfer structure, substantially contributes to PLQY enhancement.^[^ ^33^, ^34^, ^35^ ^]^

The stabilities and optical and/or electrical properties of perovskite solutions or films can be improved through the addition of quantum dots or nanocrystals (NCs).^[^ ^36^, ^37^, ^38^, ^39^ ^]^ Colloidal NCs (such as PbS or CsPbBr_3_) capped with surface‐passivating ligands can be deposited as additional passivating layers on top of 3D perovskite films to increase their operational stability in optoelectronic devices. Additionally, the in situ formation of 0D Cs_4_PbBr_6_ and 3D CsPbBr_3_ enables the surface modification of CsPbBr_3_ to enhance its PL emission and ambient stability.^[^ ^40^ ^]^ However, the exact role of 0D Cs_4_PbBr_6_ and the concurrent formation of lower‐dimensional perovskites remain unexplored despite these hybrid heterostructures providing additional freedom to enhance structural stability by replacing the labile organic ligands.

The incorporation of alkali metal halides (e.g., CsX) into lead halide perovskites has been used to enhance their structural and optoelectronic stabilities.^[^ ^41^, ^42^, ^43^, ^44^, ^45^, ^46^, ^47^ ^]^ The inorganic Cs^+^ cation, which is smaller than MA and FA, can act as an A‐site cation of the halide perovskite structure to control the tolerance factor and enhance crystal stability through octahedral distortion and tilting.^[^ ^48^, ^49^, ^50^, ^51^, ^52^ ^]^ CsX can be integrated into perovskite films using solution‐based methods to increase device stability and performance.^[^ ^41^, ^42^, ^43^, ^44^, ^45^ ^]^ For example, the addition of CsCl can increase the grain size and crystallinity of such films, leading to enhanced power conversion efficiencies in solar cells.^[^ ^41^, ^42^ ^]^ Similarly, the incorporation of CsBr can enhance the stability and performance of perovskite LEDs and solar cells.^[^ ^43^, ^44^, ^45^ ^]^ However, the direct treatment of perovskite materials with CsX solutions is challenging because of the low solubility of CsX in nonpolar solvents and the ability of polar solvents to damage the underlying layers.^[^ ^53^, ^54^, ^55^ ^]^ This problem can be mitigated through the introduction of presynthesized CsX NCs into perovskite films via postdeposition treatment, which has been reported to improve the efficiency and stability of perovskite solar cells via defect passivation and energy‐level alignment due to halide exchange.^[^ ^46^, ^47^ ^]^ Although the incorporation of CsX NCs into perovskite materials is known to enhance their stability and optoelectronic properties, the application of these NCs to quasi‐2D perovskite structures is underexplored, and the underlying mechanisms and specific role of the NCs in the structural modification of quasi‐2D and 3D perovskites are not fully understood. Further investigations are needed to elucidate these interactions, which are crucial for optimizing the performance of perovskite‐based optoelectronic devices. Therefore, a comprehensive understanding of surface reconstruction processes and their impact on optoelectronic properties is essential for rational device design and optimization.^[^ ^4^, ^27^, ^34^, ^56^ ^]^

To bridge the earlier gap, we herein systematically investigate the effects of CsX NC treatment on the optoelectronic properties and structure of quasi‐2D perovskite films, elucidating the complex interplay between halide exchange and phase transformations at the NC–film interfaces through in situ spectroscopic analyses and temperature‐dependent studies. Our findings reveal that the choice of X plays a critical role in determining the dominant structural modification pathway, with CsCl and CsI NCs preferentially inducing halide exchange and CsBr NCs inducing a phase transformation to 0D Cs_4_PbBr_6_. By controlling the reaction conditions and NC composition, we realize bandgap tuning and increase the luminescence efficiency of quasi‐2D perovskite films over a wide range of the visible spectrum, providing a new perspective on surface reconstruction in quasi‐2D perovskites.

## 2 Results and Discussion

### 2.1 Properties of CsX NCs

**Figure 1:**
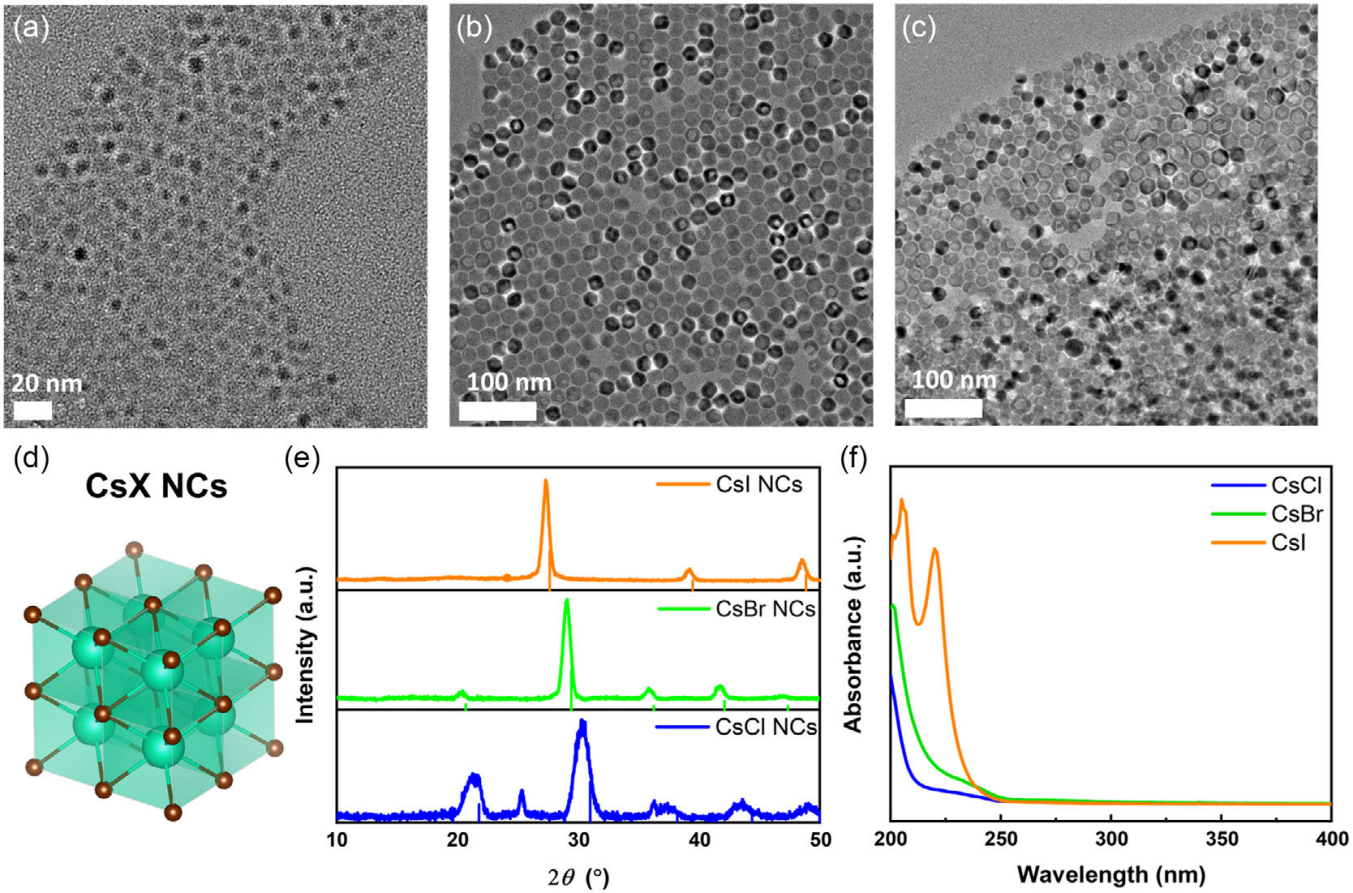
(a–c) TEM images of the CsX NCs: a) *X* = Cl, b) *X* = Br, and c) *X* = I. d) Crystal structure of the cubic‐phase CsX NCs. e) XRD patterns of the CsX NCs and corresponding references from the Inorganic Crystal Structure Database (CsCl: 01‐086‐3784, CsBr: 01‐082‐9638, and CsI: 01‐071‐5197). f) UV absorbance spectra of the CsX NCs.

Based on the results of transmission electron microscopy (TEM) imaging (**Figure** **1**a–c), the average sizes of the CsCl, CsBr, and CsI NCs were determined as 6.7 ± 0.9, 18.8 ± 1.5, and 17.0 ± 3.0 nm, respectively (Figure S1, Supporting Information). The NC size distribution could be controlled by modulating the synthesis temperature (50–80 °C). The uniform size distribution and small size of the CsX NCs enabled their even distribution on the film surface and consistent reactions at the interface. In the lattices of the CsX NCs (Figure 1d), each Cs^+^ cation is coordinated by eight X^−^ anions and vice versa.^[^ ^57^, ^58^, ^59^ ^]^ The corresponding X‐ray diffraction (XRD) patterns (Figure 1e) confirmed the presence of the cubic phase. With the increasing size of X^−^, the diffraction peaks shifted to smaller angles, in accordance with Bragg's equation. Figure 1f presents the UV–vis absorption spectra of the CsX NCs. A band‐edge absorption peak at 225 nm was observed only for the CsI NCs, while the CsBr and CsCl NCs exhibited larger bandgaps and insulating properties, with their first absorption peaks observed below 200 nm.^[^ ^57^, ^60^ ^]^

### 2.2 Surface Treatment of Quasi‐2D Film with CsX NCs (*X* = Cl, Br, and I)

**Figure 2:**
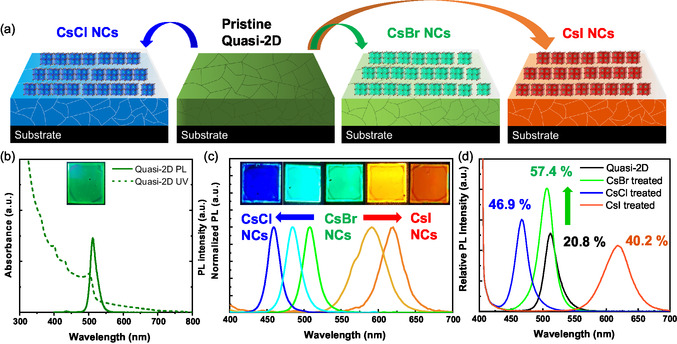
a) Schematic of the surface treatment of the quasi‐2D perovskite films with the CsX NCs. b) UV–vis absorption and PL emission spectra of the pristine quasi‐2D perovskite film. c) PL emission spectra of CsX NC‐treated quasi‐2D perovskite films and d) corresponding normalized PL emission spectra acquired using excitation at 400 nm. Insets in (b,c) show digital photographs taken under excitation at 365 nm. With the exception of (d), all PL emission spectra were recorded using excitation at 365 nm.

The construction of the CsX/quasi‐2D perovskite film heterointerfaces is illustrated in **Figure** **2**a, and the optical absorption and PL emission spectra of a representative pristine quasi‐2D perovskite film (control) are presented in Figure 2b. The absorption spectrum exhibited peaks at 400, 431, and 502 nm corresponding to 2D phases with *n* = 1, 2, and ≥5, respectively.^[^ ^61^, ^62^ ^]^ The PL emission spectrum featured a peak at 512 nm corresponding to green emission. The addition of CsCl and CsI NCs resulted in continuous film color changes (green to blue and yellowish‐orange, respectively; inset of Figure 2c) and a PL emission shift to shorter (450 nm) and longer (620 nm) wavelengths, respectively (Figure 2c and S2, Supporting Information). The halide exchange extent (or emission peak shift) depended on the NC concentration. For instance, blue PL emission at 459 nm was observed after treatment with 8 mg mL^−1^ CsCl (10 μL; 47.5 mM), and orange‐red PL emission at 618 nm was observed after treatment with 12 mg mL^−1^ CsI (10 μL; 46.2 mM). In addition, a notable PLQY increase (20.8% (pristine) < 40.2% (CsI) < 46.9% (CsCl) < 57.4% (CsBr)) was observed for all treated quasi‐2D perovskite films (Figure 2d). As a representative example, we captured a digital photograph during the treatment with the CsX NCs (Figure S3 and Video S1, Supporting Information). The earlier results suggest that the CsX NCs improved the PLQY of the pristine quasi‐2D perovskite film by passivating its surface defect states. To rule out PLQY enhancement due to the solvent effect, we treated the pristine quasi‐2D perovskite film with *n‐*hexane (10 μL), which was used to disperse the NCs. The atomic force microscopy images, UV–vis absorption spectra, and PL emission spectra collected before and after solvent addition (Figure S4, Supporting Information) revealed minimal impacts on the film surface morphology and spectroscopic features, confirming that the observed changes were predominantly due to the CsX NCs rather than the solvent.

### 2.3 In Situ Observation of Structural Changes in CsX NC‐Treated Films

**Figure 3:**
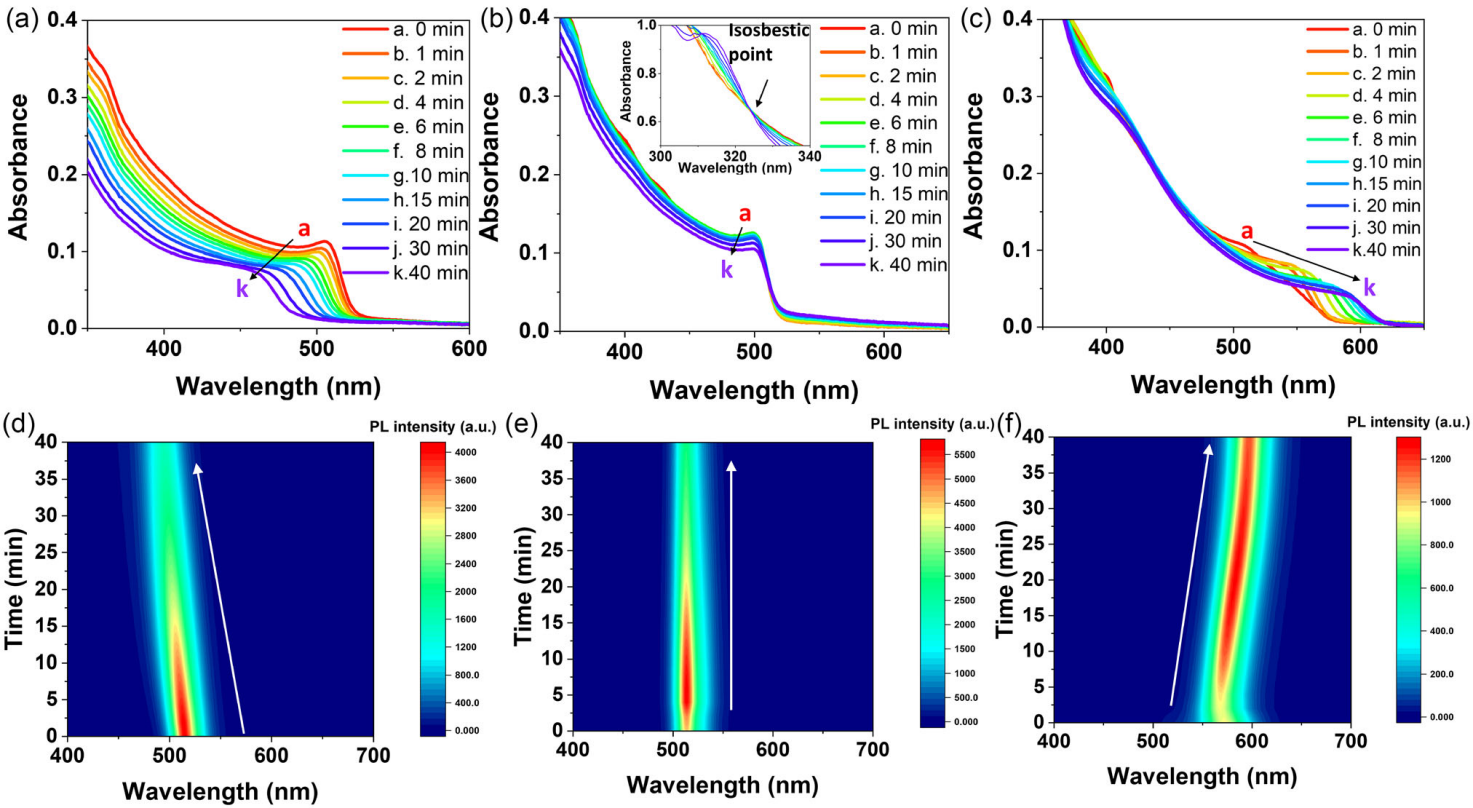
In situ a–c) absorption and d–f) PL emission spectra recorded during the ambient‐condition (≈22 °C, air) surface treatment of quasi‐2D perovskite films deposited on glass substrates with (a,d) CsCl, (b,e) CsBr, and (c,f) CsI NCs (10 μL, dispersion in n‐hexane).

The structural changes in the CsX NC‐treated quasi‐2D perovskite films spin‐cast on a glass substrate were monitored by in situ absorption (**Figure** **3**a–c) and PL emission (Figure 3d–f, Supporting Information) spectroscopy over 40 min under ambient conditions (22 °C, air). As expected from Figure 2a, the addition of the CsCl and CsI NCs induced halide exchange across the interface (or the film surface) and band‐edge excitonic absorption peak shifts from 505 nm to 470 and 595 nm, respectively. These observations suggest that halide exchange instantaneously occurred at the NC/film interface even at room temperature. The exchange was markedly faster for CsI than for CsCl, as the excitonic absorption peak immediately shifted to longer wavelengths (520–550 nm) in the former case (Figure 3c and S2a, Supporting Information).

The iodide‐induced PL broadening is attributable to an inhomogeneous Br ↔ I exchange that generates I‐rich (or high‐*n*) subdomains with smaller bandgaps. Such mixed‐halide inhomogeneity is well‐known to yield dual or broadened emission bands in CsPb(Br/I)_3_ systems.^[^ ^21^, ^61^, ^63^, ^64^, ^65^ ^]^ In our films, this manifests as a new shoulder at 560–570 nm (Figure 2c), whereas CsBr or CsCl treatments—which do not introduce a new low‐bandgap halide phase—preserve the narrow PL profile.

The addition of CsBr NCs did not induce halide exchange because the perovskite film contained bromide ions, and the position of the band‐edge absorption peak (505 nm) therefore did not change. The magnification of the 300–340 nm region (inset of Figure 3b) revealed an isosbestic point at 325 nm, which indicated the occurrence of a chemical transformation. The intensification of the absorption at 315 nm with time corresponded to the formation of Cs_4_PbBr_6_ NCs and suggested that CsBr transformed into Cs_4_PbBr_6_ at the expense of quasi‐2D lead halide perovskites. The CsBr layer was very Pb‐deficient, whereas the quasi‐2D perovskite layer was relatively Pb‐rich, thus acting as a Pb source. The Pb equilibrium could induce the structural phase transformation from CsBr to Cs_4_PbBr_6_ (0D phase).

Such structural transformations (including halide exchanges with Cl and I or the phase transformation with Br) could be better visualized using in situ PL emission spectroscopy (Figure 3d–f). As indicated by the absorption changes, the initial PL emission maximum of the quasi‐2D perovskite films (515 nm) shifted to shorter (490 nm) and longer (600 nm) wavelengths in 40 min following CsCl and CsI treatments, respectively (Figure 3d,f). In contrast, the addition of CsBr did not cause any peak shift (Figure 3e). Figure S5, Supporting Information, summarizes the time‐dependent evolution of the band‐edge emission peak for these films. Notably, the PL emission intensity of the pristine quasi‐2D perovskite film initially increased 1.2–1.5‐fold after 5 min, subsequently decreasing (Figure S6, Supporting Information). The initial PL intensity increase was associated with the NC‐induced surface reconstruction and passivation. However, the ambient air present during the surface modification and reconstruction degraded the PL emission properties of the films, as the infiltration of oxygen and moisture introduced defects and nonradiative recombination centers.^[^ ^66^, ^67^ ^]^ When the same experiment was performed in an O_2_‐free glovebox, the PLQY continuously increased (Figure 2d). We also measured PL decay lifetimes after NC treatments using time‐correlated single photon counting (Figure S7, Supporting Information). Using tri‐exponential fitting of the decay curves presented in Table S1, Supporting Information, we can deconvolute the contribution of fast, intermediate, and slower components that can be assigned for the trapping (decreased weighting), radiative (increased weighting), and nonradiative decay processes, respectively. As expected, the average PL lifetime of the pristine quasi‐2D perovskite film (11.9 ns) decreased to 5.7 ns upon treatment with CsBr as a result of surface passivation with 0D Cs_4_PbBr_6_ layer atop quasi‐2D perovskites. Upon treatment with CsCl and CsI NCs, respectively, the average lifetime changed to 6.4 ns (CsCl‐treated), and to 20.1 ns (CsI‐treated) owing to halide ion‐dependent charge carrier recombination dynamics.^[^ ^10^, ^68^, ^69^ ^]^

### 2.4 Observation of Surface Changes after Treatment with CsX NCs

**Figure 4:**
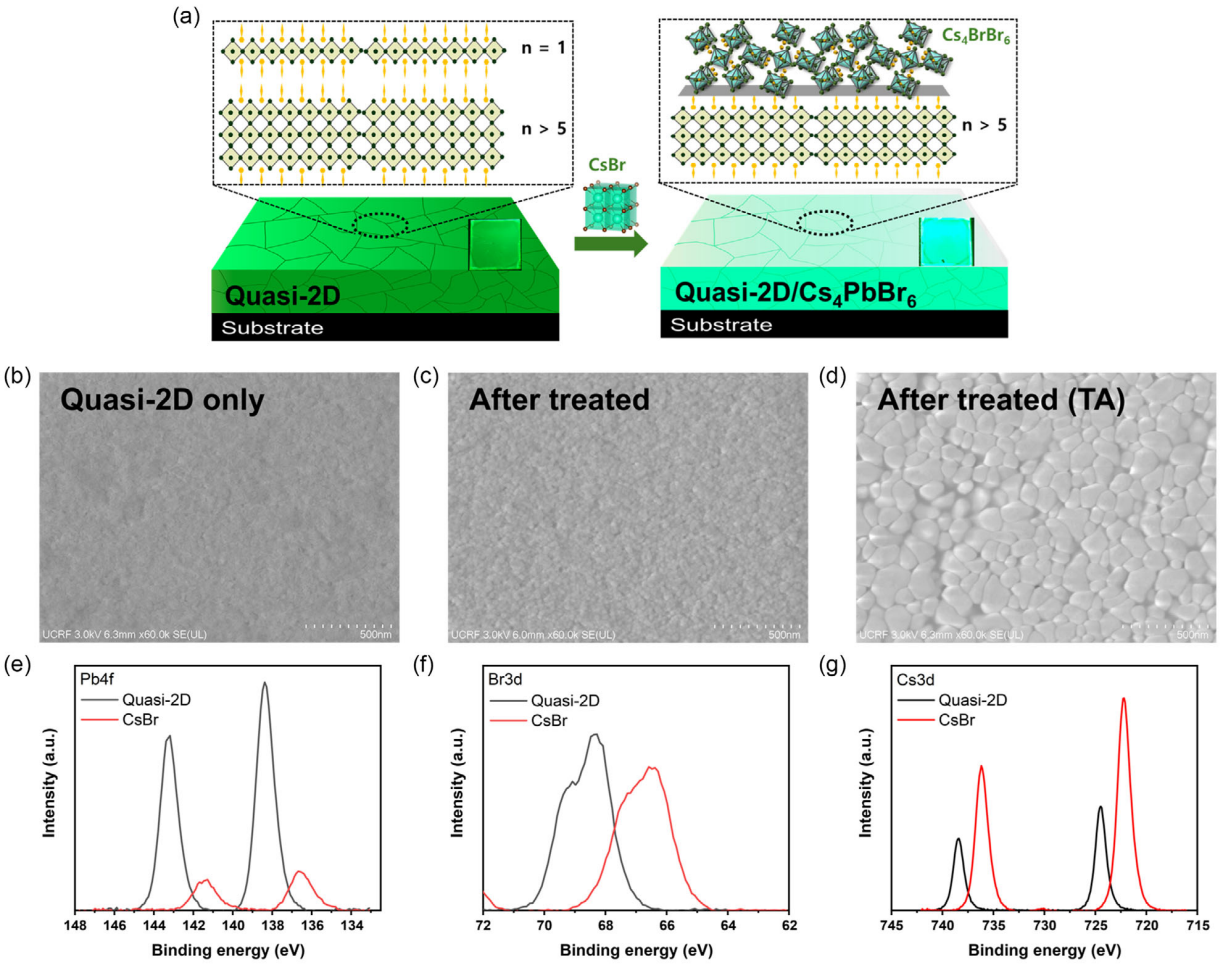
a) Schematic of the structural phase transformation in the CsBr NC/quasi‐2D perovskite film system. SEM images of b) the pristine quasi‐2D perovskite film, c) film treated with the CsBr NCs, and d) film treated with the CsBr NCs and then thermally annealed. e) Pb 4*f*, f) Br 3*d*, and g) Cs 3*d* spectra of the CsBr NC‐treated film.

The phase transformation induced by the CsBr NCs was probed by scanning electron microscopy (SEM) and X‐ray photoelectron spectroscopy (XPS). **Figure** **4**a illustrates the phase transformation occurring at the CsBr/quasi‐2D perovskite film interface. The only compositional difference between the CsBr NCs and quaternary PEA–Cs–Pb–Br film was the presence of Pb, which suggested that Pb^2+^ ions tended to be in chemical equilibrium across the interface. Figure 4b shows that the pristine quasi‐2D perovskite film had a uniform grain size of ≤20 nm. The addition of the CsBr NCs (10 mg mL^−1^; 47 mM) resulted in their uniform distribution throughout the film (Figure 4c). Complete NC coverage was observed for undiluted to twofold diluted (with *n*‐hexane) NC solutions, and a coverage of 52.11% was observed for eightfold dilution (Figure S8, Supporting Information). To accelerate the reaction caused by the CsBr NC treatment, additional thermal annealing (60 °C, 5 s) was conducted, with the resulting interfacial morphology changes shown in Figure 4d. Annealing substantially changed the average grain size, which was determined as 110.77 ± 25.34 nm, and the NC coverage was determined as 100%. The extent of this change depended on X (Figure S9, Supporting Information). In the case of the CsI NCs, the film morphology did not markedly change after additional annealing. In contrast, for the CsCl NC‐treated samples, large grains (>400 nm) were formed even without annealing. This trend resembled the reported tendency of chloride (e.g., CsCl)‐containing perovskites to exhibit larger grain sizes than iodide‐only perovskites.^[^ ^41^, ^42^, ^49^ ^]^

The surface compositional changes induced by the CsX NC treatments were further investigated using XPS. The Pb 4f spectrum of the pristine quasi‐2D perovskite film featured two prominent peaks at 138.3 eV (Pb 4*f*_7/2_) and 143.2 eV (Pb 4*f*_5/2_) (Figure 4e). After the treatment with the CsBr NCs, the Pb 4f peaks considerably weakened and (together with the Br 3 d and Cs 3*d* peaks) shifted to lower binding energies (Figure 4e–g). These changes indicated a surface transformation from a Pb‐rich environment to a Cs‐rich one. The XPS data of the CsBr NC‐treated films deviated from those typically reported for conventional perovskites,^[^ ^33^, ^70^, ^71^ ^]^ suggesting structural changes rather than simple variations in bonding and resembling data reported for CsX and Cs_4_PbX_6_ structures.^[^ ^72^, ^73^, ^74^ ^]^ The Br 3*d* peaks at 66.4 and 67.2 eV were ascribed to isolated [PbBr_6_]^4−^ ions within the Cs_4_PbBr_6_ structure or the CsBr lattice.^[^ ^75^ ^]^ Furthermore, the emergence of Cl 2*p* and I 3*d* peaks after treatments with the CsCl and CsI NCs, respectively, indicated the occurrence of halide exchange on the surface (Figure S10, Supporting Information).

### 2.5 Mechanistic Understanding of Structural Changes: Halide Exchange (*X* = Cl, I) versus Phase Transformation (*X* = Br)

**Figure 5:**
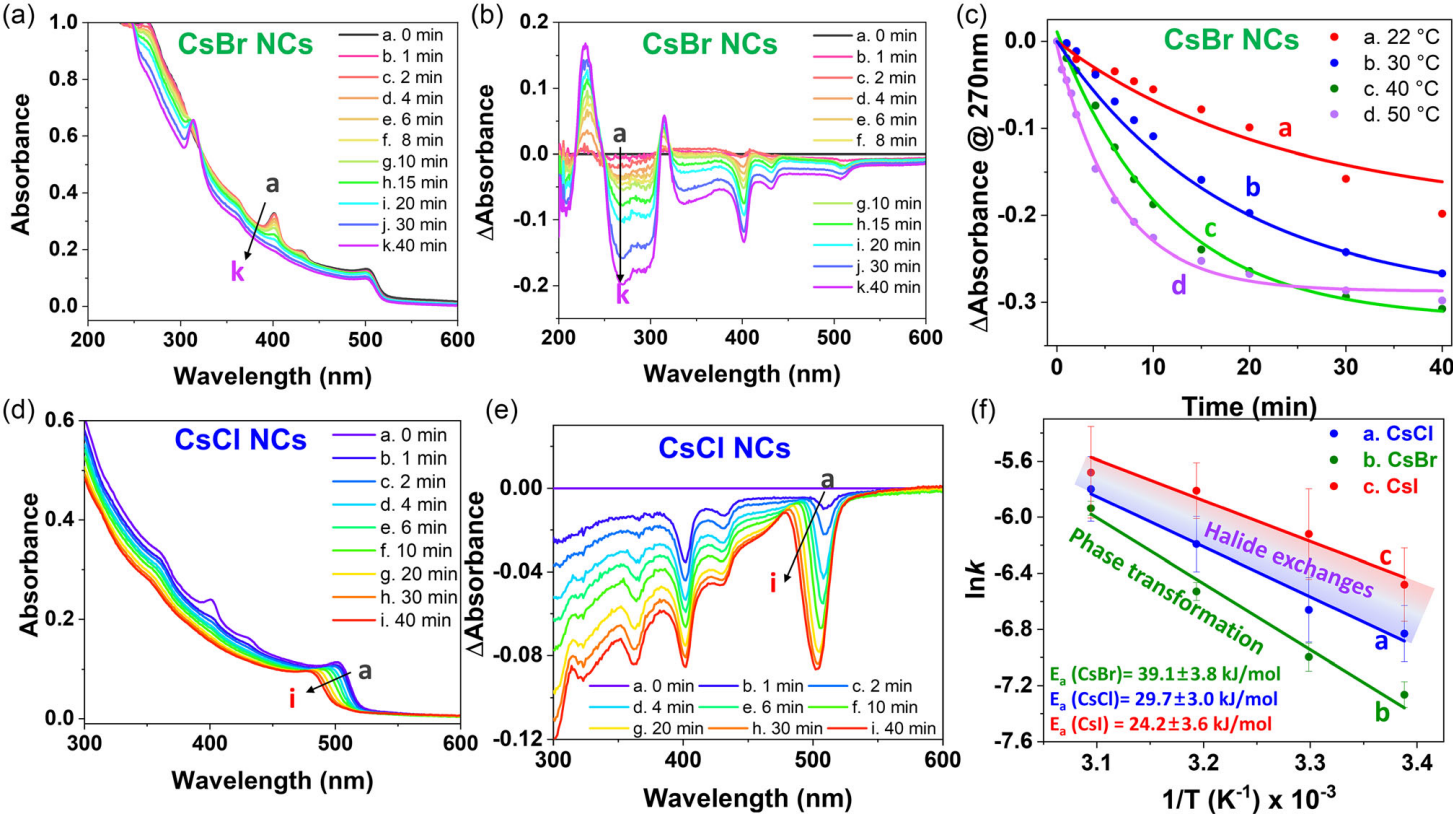
a,b) UV–vis absorption and difference absorption spectra of CsBr NC‐treated quasi‐2D perovskite films on quartz recorded at 22 °C. c) Corresponding kinetic traces and fits obtained by tracking the absorption at 270 nm in (b) at 22–50 °C. d,e) UV–vis absorption and difference absorption spectra of CsCl NC‐treated quasi‐2D perovskite films on quartz recorded at 22 °C. f) Plots of ln(*k*) versus 1/*T* for surface treatments with the CsX NCs. Note that monoexponential fits were used for rate constant (*k*) determination.

To further investigate structural changes, including the phase transformation upon the addition of the CsBr NCs or halide exchange upon the addition of the CsCl and CsI NCs, we characterized quasi‐2D perovskite films CsX‐treated at different temperatures (22–50 °C) on quartz substrates. Previously, 2D perovskite films were obtained on glass substrates, which precluded spectroscopic characterization at wavelengths below 350 nm because of light scattering or absorption.^[^ ^30^, ^76^ ^]^ Hence, quartz was used to unveil time‐dependent spectral changes and minimize potential spectral interferences in this region. **Figure** **5**a shows the temporal absorption changes recorded for CsBr/quasi‐2D perovskite films on quartz at room temperature (22 °C), revealing an isosbestic point at 315 nm and a decrease in the band‐edge absorption intensity from 0.15 at 0 min to 0.10 at 40 min. As shown earlier, thinner and terminal layers of quasi‐2D perovskite films with *n* = 1 (as demonstrated in Figure 4a) underwent structural phase transformations according to Equation (1).

(1) $$\text{4CsBr }\left(\text{Pb‐deficient}\right){\text{ + PEA}}_{2}{\text{PbBr}}_{4}\left(\text{Pb‐rich}\right)\to {\text{Cs}}_{4}{\text{PbBr}}_{6}\text{+ 2PEABr}$$

The quasi‐2D perovskites with PEA_2_PbBr_4_ (*n* = 1) as the terminal layer were probably the first to react with the CsBr NCs to yield Cs_4_PbBr_6_ owing to Pb chemical potential difference across the CsX NCs and quasi‐2D film.^[^ ^77^, ^78^ ^]^ Such spectroscopic changes are better visualized using difference absorption spectra (Δ*A*; Figure 5b), with positive (negative) absorption suggesting the formation (consumption) of new (old) species. The new peak at 315 nm corresponded to the in situ generation of Cs_4_PbBr_6_, while the first, second, and third band‐edge peaks at 400, 430, and 500 nm corresponding to *n* = 1, 2, and thicker, respectively, lost intensity. By tracking the decrease in the absorption at 270 nm, which represents the overall consumption of the quasi‐2D perovskite, we determined the kinetics of the phase transformation. The same experiment was conducted at elevated temperatures (30, 40, and 50 °C; Figure S11, Supporting Information). Figure 5c shows the kinetic traces and fits (270 nm) corresponding to the consumption of the parent quasi‐2D perovskite films at 22–50 °C. Monoexponential fitting was used to determine the kinetic rate constant (*k*) at different temperatures. Interestingly, the consumption of the parent 2D films (plus the phase transformation to Cs_4_PbBr_6_) was accelerated at higher temperatures. Specifically, *k* increased from 7.0 × 10^−5^ s^−1^ at 22 °C to 2.6 × 10^−3^ s^−1^ at 50 °C, that is, by two orders of magnitude. As reported previously, the connectivity of Cs^+^ ions in CsBr (cubic) can be easily altered to afford Cs_4_PbBr_6_ (hexagonal) upon the insertion of Pb^2+^ ions.^[^ ^4^, ^57^, ^58^ ^]^

To understand the preferential occurrence of halide exchange (and not structural transformations) upon CsCl and CsI treatments, we conducted similar experiments with CsCl and CsI at different temperatures (22–50 °C) (Figure S12, S13, Supporting Information) to induce halide exchange, with a representative example for halide exchange with CsCl at 22 °C provided in Figure 5d and e. In these cases, the first excitonic peak of the quasi‐2D perovskite films shifted to shorter wavelengths for CsCl and longer wavelengths for CsI because of the formation of a mixed halide phase across the interface. The difference in absorption spectra directly indicated the formation of mixed‐halide quasi‐2D perovskite films. By conducting the same experiments at 22–50 °C, we quantified the reaction kinetics. For halide exchange, the band‐edge absorption peak was tracked to determine the rate constant (*k*) by monoexponential fitting (Figure S14, Supporting Information). The Arrhenius (ln(*k*) vs. 1/*T*) plots for the CsX NC‐induced structural changes, that is, phase transformation to Cs_4_PbBr_6_ (CsBr NCs) and halide exchange (CsCl and CsI NCs), are presented in Figure 5f. The activation energy for the CsBr NC‐induced phase transformation (39.1 ± 3.8 kJ mol^−1^) exceeded that for the CsCl and CsI NC‐induced halide exchange (24–29 kJ mol^−1^). The activation energy for the halide exchange was associated with the Pb–X bond energy, as described elsewhere.^[^ ^21^, ^79^ ^]^ These findings suggested that halide exchange across the interface was kinetically favored.

**Figure 6:**
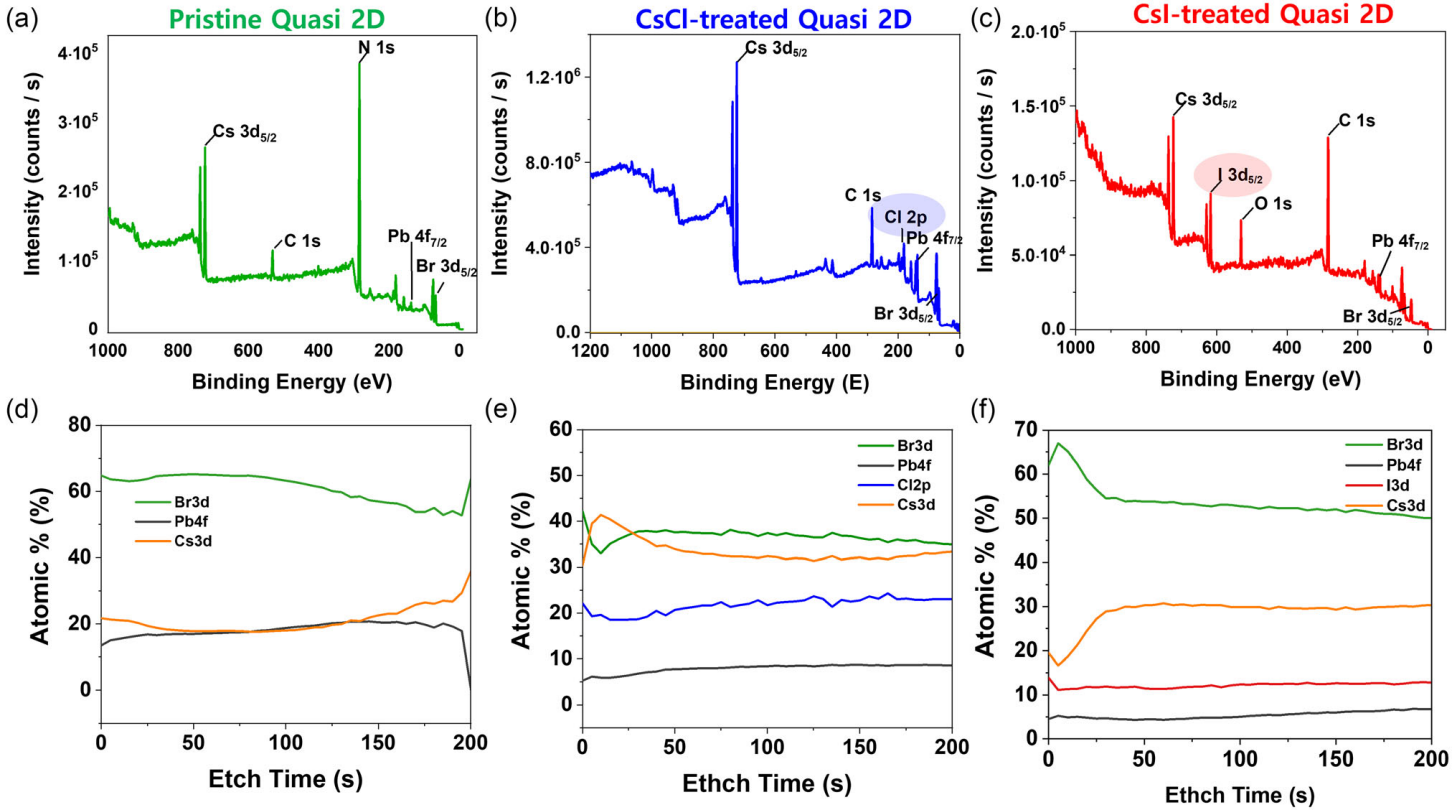
a–c) XPS survey scan analysis for pristine quasi‐2D perovskite (a), CsCl NC‐treated quasi‐2D (b), and CsI NC‐treated quasi‐2D perovskite film (c), respectively. d–f)Corresponding in‐depth XPS analysis of each film after etching at a rate of 0.26 nm s^−1^ (with reference to Ta_2_O_5_).

To further understand the halide exchange of quasi‐2D perovskite films with CsCl and CsI NC treatments, respectively, we have analyzed the halide composition of pristine and CsX NC‐treated films using XPS. The XPS survey scan as well as XPS spectra of each halide (Cl 2*p*, Br 3*d*, and I 3*d*) revealed that CsX NC (*X* = Cl and I) treatment of quasi‐2D perovskite induced the halide exchange reaction across the interface of NC and quasi‐2D film due to the presence of Cl 2*p* and I 3*d* peaks after treatment (**Figure** **6**a–c and Figure S10, Supporting Information). The XPS depth profiles for each elemental composition were obtained through sequential X‐ray ion beam etching of the film. The sputter rate regarding Ta_2_O_5_ was known as 0.26 nm s^−1^. The XPS depth profile changes as a function of etching time demonstrated that the pristine quasi‐2D perovskites were characterized by a homogenous distribution of Cs:Pb:Br with an approximate stoichiometric ratio of 1:1:3. In stark contrast, upon treatment of the quasi‐2D perovskite films with CsCl or CsI NCs, newly appeared Cl 2*p* and I 3*d* peak intensities can be initially observed with their atomic contribution percent across the film thickness maintained. These reflect that the halide ions (Cl and I) were homogeneously mixed and distributed across the film. The rationale behind the decreased Pb concentration for the CsX NC‐treated quasi‐2D perovskites compared to the pristine film is mainly due to the incorporation of CsX composition into the film.

The XRD analyses of films 12 h after treatments with the CsCl and CsBr NCs revealed the formation of Cs_4_PbCl_6_ and Cs_4_PbBr_6_, respectively (Figure S15, Supporting Information). Once the halide exchange reached equilibrium, the phase transformation to Cs_4_PbX_6_ could be sequentially activated to attain a Pb equilibrium across the interface.

### 2.6 Fabrication and Characterization of Red–Green–Blue (RGB) LEDs

**Figure 7:**
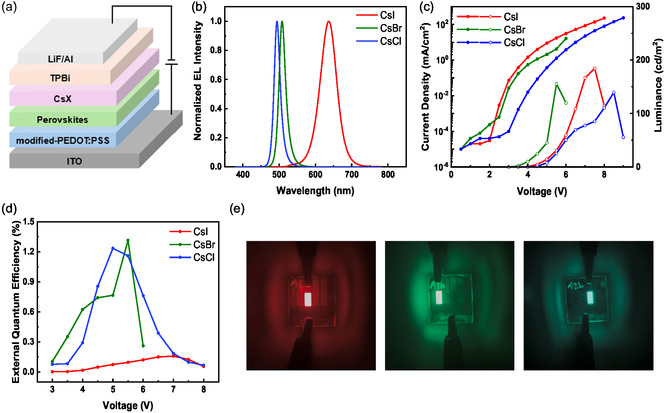
a) Schematic of CsX NC‐treated quasi‐2D PeLEDs. b) EL spectra recorded after PeLED treatment with the CsX NCs. c) Current density–voltage–luminance (*J*–*V*–*L*) plots of PeLEDs. d) PeLED external quantum efficiency as a function of voltage. e) Digital photographs taken during the operation of red, green, and sky‐blue PeLEDs.

To showcase the practical applications of the surface reconstruction of the quasi‐2D perovskite films induced by the CsX NCs, we fabricated perovskite LEDs (PeLEDs) using the modified films as emissive layers, with the device structure corresponding to indium tin oxide (ITO)/modified poly(3,4‐ethylenedioxythiophene):polystyrene sulfonic acid (PEDOT:PSS)/perovskite/CsX NCs/1,3,5‐tris(*N*‐phenylbenzimidazol‐2‐yl) benzene (TPBi)/LiF/Al (**Figure** **7**a).

The electroluminescence (EL) spectra of PeLEDs treated with CsI, CsBr, and CsCl NCs (2.5 mg mL^−1^) displayed emission peaks at 637, 508, and 494 nm, respectively (Figure 7b), which demonstrated that the emission wavelength could be tuned through the purposeful choice of X. The CIE 1931 chromatic coordinates of the CsI, CsBr, and CsCl NC‐treated PeLEDs, namely (0.6362, 0.3523), (0.0721, 0.6978), and (0.053, 0.415), respectively, further confirmed color tunability (Figure S16, Supporting Information). The full width at half‐maximum values of the EL peaks of the CsBr‐ and CsCl‐treated devices (23.76 and 21.73 nm, respectively) were comparable to that of the device (control) fabricated without the CsX NC treatment (Figure S17, Supporting Information).

The results of current density–voltage–luminance (*J*–*V*–*L*) characterization revealed that the maximum luminance values observed for the devices treated with the CsI, CsBr, and CsCl NCs were 184.57, 155.38, and 139.6 cd m^−2^, respectively (Figure 7c). The external quantum efficiencies (EQEs) of the NC‐treated PeLEDs are shown in Figure 7d, and the photographs in Figure 7e confirm the successful modulation of emission color through surface treatment. The performances of control and CsX‐treated devices are summarized in Figure S17 and Table S2, Supporting Information. And the EQE statistics histograms of the CsX‐treated devices are shown in Figure S18, Supporting Information, which reveals a similar performance for each type of surface treatment.

We also have analyzed the carrier dynamics using electrochemical impedance spectroscopy (EIS). The equivalent circuit used for fitting is shown in the inset of Figure S19, Supporting Information. In Figure S19, Supporting Information, the control device exhibited the lowest diameter of the semicircle in EIS spectra, which means the lower recombination resistance compared to CsX‐treated PeLEDs, confirming the highest EQE value in device performance.^[^ ^80^, ^81^ ^]^ In addition, the carrier transport constant for the device with higher EQE was shorter (*τ*_CT_ = 1.97, 4.57, 3.10, and 2.70 μs for the control, CsI, CsBr, and CsCl‐treated devices, respectively). The deteriorated carrier transport and recombination characteristics, and the lower performance, could be attributed to the insulating nature of the CsX NCs.

To further demonstrate the possibility of achieving pure RGB emission, we optimized LED fabrication by varying the NC concentration. Specifically, 5.0 mg mL^−1^ CsI, 2.5 mg mL^−1^ CsBr, and 7.5 mg mL^−1^ CsCl NC solutions were used for surface treatment. The resulting PeLEDs featured EL peaks at 649, 508, and 464 nm, respectively, which confirmed the realization of RGB emission, and Figure S20, Supporting Information, illustrates the color space expansion induced by surface treatment.

## 3 Conclusion

The optoelectronic properties of quasi‐2D perovskite films were efficiently modulated through surface treatment with CsX (*X* = Cl, Br, I) NCs, which resulted in halide exchange (*X* = Cl, I) or a structural transformation at the NC/quasi‐2D perovskite interface (*X* = Br). These surface reconstruction processes enabled the tuning of the optical bandgap and PL properties across the visible spectrum (450–620 nm). The underlying mechanisms and kinetic favorability of the competing reaction pathways were examined using in situ spectroscopic techniques and temperature‐dependent kinetic studies. The activation energy for halide exchange (24–29 kJ mol^−1^) was lower than that for the structural phase transformation to 0D Cs_4_PbX_6_ NCs (39 kJ mol^−1^), which indicated a kinetic preference for the former process. To showcase the practical potential of our approach, we fabricated LEDs with CsX NC‐treated quasi‐2D perovskite films as the emission layer. By optimizing the NC type and concentration, we achieved red, green, and blue EL. These results underscore the potential of CsX NC treatment as a versatile strategy for developing spectrally diverse perovskite‐based optoelectronic devices. Our work sheds light on the complex interplay between the composition, structure, and properties of quasi‐2D perovskites, providing a versatile tool for the systematic design and optimization of such devices. The ability to selectively trigger halide exchange or phase transformation through straightforward postsynthesis treatments opens new possibilities for the design of perovskite‐based devices with tailored properties and enhanced performance. We believe that the insights gained from this study will stimulate further research in the field of perovskites and contribute to the advancement of optoelectronic technologies.

## 4 Experimental Section

4.1

4.1.1

##### Materials

ZnBr_2_ (99.999%), ZnI_2_ (98%), InCl_3_ (99.999% trace metals basis), CsBr (99.999% trace metals basis), PbBr_2_ (98% trace metals basis), Cs_2_CO_3_ (99.9%), phenethylammonium bromide (PEABr, 98%) 1‐octadecene (ODE, technical grade, 90%), oleylamine (OAm, 70%), oleic acid (OA, 90%), *n*‐hexane (anhydrous, 95%), and dimethyl sulfoxide (DMSO, anhydrous, 99.9%) were purchased from Sigma Aldrich. TPBi (99.9%) was purchased from OSM. All chemicals were used as received.

##### Preparation of Cesium Oleate

Cs_2_CO_3_ (0.4 g) was mixed with OA (8 mL), and the mixture was heated at 150 °C for 30 min using a hotplate. The molten cesium oleate was held at 120 °C using the same hotplate up to the point of injection.

##### Synthesis and Purification of the CsX NCs

The CsX NCs were synthesized using a modification of a previously reported method.^[^ ^57^, ^82^ ^]^ The halide precursor (0.1 mmol; ZnBr_2_ for CsBr, InCl_3_ for CsCl, and ZnI_2_ for CsI) was added to a vial (20 mL) containing ODE (5 mL), OA (0.2 mL), and OAm (1.5 mL), and the mixture was heated at 150 °C for 30 min on a hotplate, placed into an oil bath (80 °C for InCl_3_, 50 °C for ZnBr_2_ and ZnI_2_) and supplemented with cesium oleate (0.75 mL). After 30 s, the solution was cooled using an ice–water bath for 5 min and centrifuged at 4000 rpm for 10 min. The NC precipitate was dispersed in *n‐*hexane (1 mL). The dispersion was treated with methyl acetate (2 mL) to remove extra surface ligands and centrifuged at 7800 rpm for 2 min. Finally, the precipitated NCs were dispersed in *n‐*hexane (1 mL) for further treatments, with the NC concentrations determined as 8, 10, and 12 mg mL^−1^ for CsCl, CsBr, and CsI, respectively.

##### Quasi‐2D Perovskite Film Fabrication

The glass or quartz substrate (2.5 × 2.5 cm) was cleaned with soapy water, rinsed with excess ethanol, blown dry with air under ambient conditions, treated with UV/ozone for 10 min to eliminate organic residues, and immediately transferred to a nitrogen‐filled glovebox (<10 ppm O_2_). A solution (40 μL) prepared by dissolving CsBr (0.3 mmol), PEABr (0.15 mmol), and PbBr_2_ (0.3 mmol) in DMSO (1 mL), followed by overnight stirring at 30 °C, was dynamically drop‐cast onto the preheated substrate (60 °C), followed by spin coating at 4000 rpm for 60 s at an acceleration of 1300 rpm. Ethyl acetate (0.5 mL) was rapidly injected as an antisolvent after 30 s. After spin casting was stopped, the perovskite film was transferred to a hotplate and annealed at 60 °C for 5 min.

##### CsX NC Treatment of Quasi‐2D Perovskite Films

A dispersion of CsX (*X* = Cl, Br, I) NCs in *n*‐hexane (10 μL) was spin cast on the quasi‐2D perovskite film, which was followed by spinning at 3000 rpm for 10 s. The film was transferred to a UV–vis spectrophotometer or spectrofluorometer for in situ or ex situ characterization.

##### Fabrication of RGB PeLEDs

An ITO patterned glass substrate was sequentially cleaned in deionized water, acetone, and isopropyl alcohol using ultrasonication and then oven‐dried overnight at 100 °C, treated with UV/ozone for 30 min, spin‐coated with the modified PEDOT:PSS solution (AI4083 mixed with L‐phenylalanine (9 mg mL^−1^)) at 4000 rpm, and annealed at 150 °C for 10 min.^[^ ^33^ ^]^ After the perovskite film fabrication, the emissive layer was coated with the NCs of choice and annealed at 60 °C for 10 min. Finally, TPBi (50 nm), LiF (1.5 nm), and Al (100 nm) were sequentially deposited via thermal evaporation under high vacuum (<10^−6^ Torr). The device active area was 8 mm^2^.

##### Characterization

Absorption spectra were recorded using a spectrophotometer (UV‐1800, Shimadzu). Time‐resolved PL measurements were carried out using a time‐correlated single‐photon‐counting setup (Fluo‐Time 300, PicoQuant). TEM imaging (JEM‐2100, JEOL) was performed at an acceleration voltage of 200 kV for samples prepared by depositing a diluted NC solution in *n*‐hexane onto a Cu grid (Ted Pella) and allowing it to dry. XRD analysis (D/MAX2500V/PC, Rigaku) was conducted at 40 kV/200 mA using Cu *K*α radiation (*λ* = 1.5405 Å). The PLQYs of perovskite films with and without CsX NCs were determined using a Quantaurus‐QY Plus UV–near‐infrared absolute PL quantum yield spectrometer (C13534‐33, Hamamatsu Corp.) equipped with a xenon lamp as the light source and a 3.3‐inch integrating sphere. For SEM imaging (SU8220, Hitachi), perovskite films with and without CsX NCs were prepared on cleaned ITO patterned glass. XPS (K‐Alpha, Thermo Fisher) analysis was performed for perovskite films with and without CsX NCs on cleaned ITO substrates.

##### In Situ Absorption Measurements

Absorption spectra were recorded using a Varian Cary 60 spectrometer in the wavelength range of 350–800 nm at a scan rate of 4800 nm min^−1^ during 40 min (interval = 30 s) at room temperature. For the variable‐temperature experiment, a single‐cell Peltier accessory (Agilent Technologies, SPV 1 × 0) was connected to the spectrometer, and spectra were recorded at 22–50 °C under the abovementioned conditions. Difference absorption (Δ*A*) spectra were acquired by subtracting the absorption spectrum at time zero (0 min; reference) from those collected at longer times.

##### In Situ PL Measurements

In situ PL measurements were conducted using a JASCO FP‐8550 spectrofluorometer in the wavelength range of 400–700 nm at a scan rate of 5000 nm min^−1^ and room temperature for 40 min (interval = 30 s). The CsX NC‐treated 2D perovskite films were excited at 365 nm, and emission spectra were collected at an interval of 0.2 nm and a slit width of 2.5 nm.

##### XPS in‐depth Analysis

XPS analysis was conducted using a Thermo Scientific K‐Alpha^+^ G2 spectrometer equipped with a monochromatic Al Kα X‐ray source (1486.6 eV) operated at 12 kV and 6 mA. A hemispherical energy analyzer was used to acquire survey scans, and the analyzer was scanned to get high‐resolution spectra in an area of 400 um (X‐ray spot size). The pass energy was 200 eV for survey scans and 50 eV (or lower) for high‐resolution scans. For depth profile measurements, Ar^+^ ion sputtering was performed using the built‐in ion source operated at 1000 eV and the high‐resolution XPS spectra were acquired from an area of 400 um for every 5 s for one cycle. A hemispherical energy analyzer scanned an area of 400 um with 50.0 eV pass energy. Charge compensation was achieved using a dual‐beam flood gun system. All binding energies were calibrated using the C 1*s* peak at 284.8 eV. The XPS analysis conducted under ultrahigh‐vacuum conditions in analysis chamber pressures maintained below 1 × 10^−^^7^ mbar.

##### EIS Measurements

EIS was conducted using a potentiostat (IviumStat) under no light conditions with frequency range from 100 Hz to 1 MHz.

##### PeLED Characterization

The *J–V*–*L* characterization of PeLEDs was performed under ambient conditions using a Keithley 2400 source measurement unit and Konica Minolta spectroradiometer (CS‐2000, Minolta Co.).

## Conflict of Interest

The authors declare no conflict of interest.

## Supporting information

Supplementary Material

## Data Availability

The data that support the findings of this study are available in the supplementary material of this article.
