# Supplementary material for: Surface Reconstruction in Quasi‐2D Perovskite Films Treated with Cesium Halide Nanocrystals: Halide Exchange or Phase Transformation
Source: Small Sci. 2025 Jul 8;5(9):2500163. doi: 10.1002/smsc.202500163 (PMC12412550; doi:10.1002/smsc.202500163)
Supplement: Supplementary file 1 — Supplementary Material [file SMSC-5-2500163-s001.zip › smsc.202500163-sup-0001-suppdata-S1.pdf]

Supporting Information

Surface Reconstruction in Quasi-Two-Dimensional Perovskite Films Treated with Cesium Halide Nanocrystals: Halide Exchange or Phase Transformation

Dong Il Son, Seonhong Min, Sohyeon An, Dongryeol Lee, Se Hyun Lee, Donghan Kim, Myoung Hoon Song, Jin Young Kim, Sungwook Park*, Junsang Cho*, and Jongnam Park**

D. I. Son, S. H. Lee, J. Park

Graduate School of Semiconductor Materials and Device Engineering, Ulsan National Institute of Science and Technology (UNIST), Ulsan 44919, Republic of Korea

E-mail: jnpark@unist.ac.kr

S. Min, J. Cho

School of Chemistry and Energy, Sungshin Women's University, Seoul 01133, Republic of Korea

E-mail: jcho3@sungshin.ac.kr

S. Park

UniQDot, Ulsan 44919, Republic of Korea

Email: s.park@unist.ac.kr

S. An, J. Y. Kim

School of Energy and Chemical Engineering, Ulsan National Institute of Science and Technology (UNIST), Ulsan 44919, Republic of Korea

D. Lee, D. Kim, M. H. Song

Department of Materials Science and Engineering, Ulsan National Institute of Science and Technology (UNIST), Ulsan 44919, Republic of Korea

J. Y. Kim

Graduate School of Carbon Neutrality, Ulsan National Institute of Science and Technology (UNIST), Ulsan 44919, Republic of Korea

J. Park

Department of Biomedical Engineering, Ulsan National Institute of Science and Technology (UNIST), Ulsan 44919, Republic of Korea

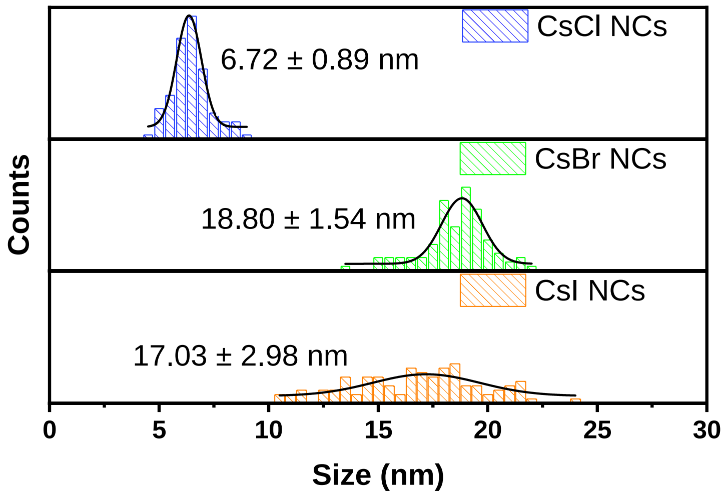

Figure S1. Size distributions of CsX (X = Cl, Br, I) nanocrystals (NCs).

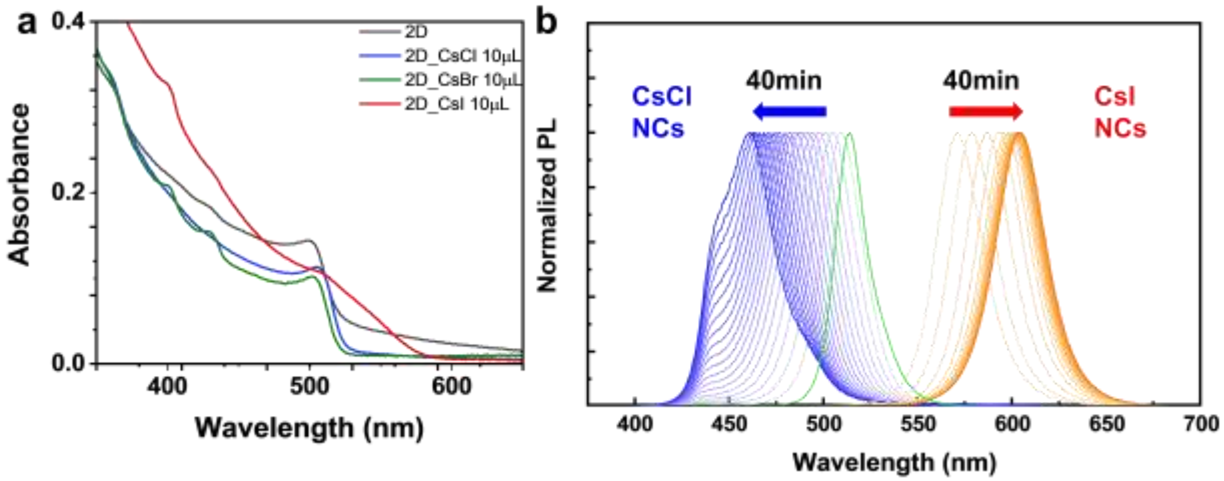

Figure S2. (a) UV–vis absorption spectra of pristine and CsX NC–treated quasi-two-dimensional (2D) perovskite films. (b) Changes in the in situ photoluminescence (PL) emission spectra observed upon the addition of CsCl and CsI NC solutions (10 μL) to the above films.

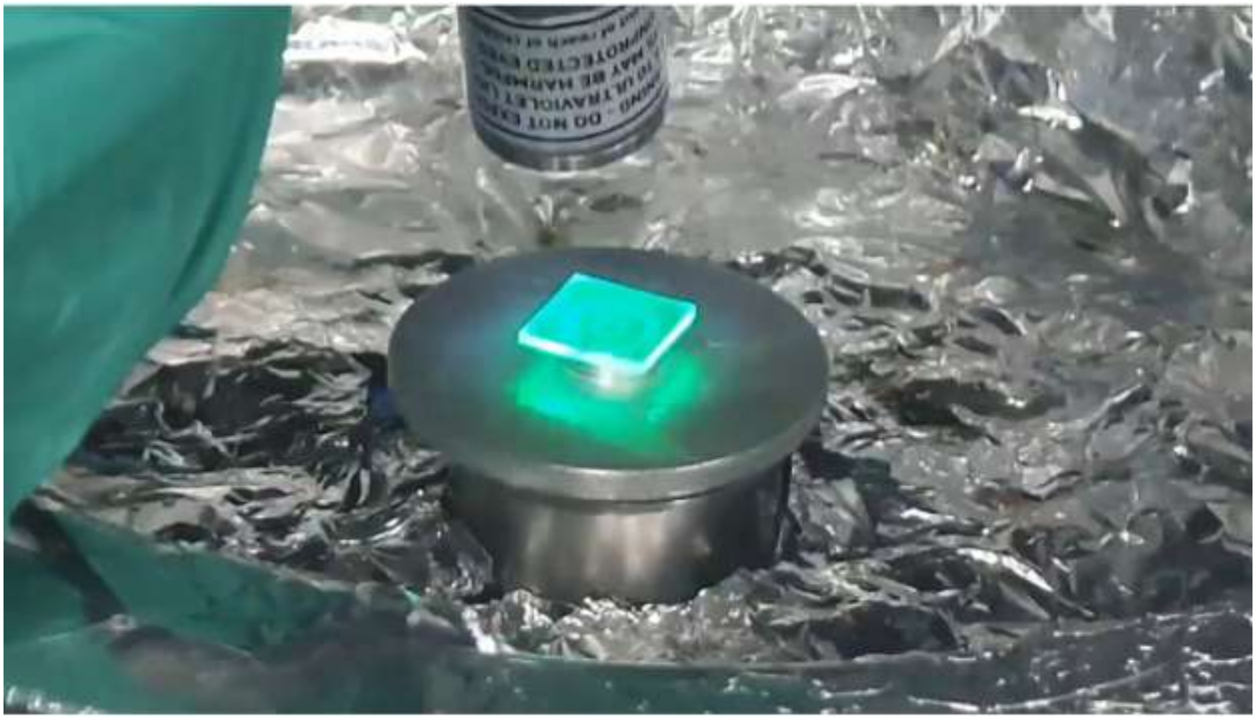

Figure S3. Digital photograph taken immediately after the addition of the CsI NCs to a quasi-2D perovskite film in a glovebox (O_2 < 10 ppm).

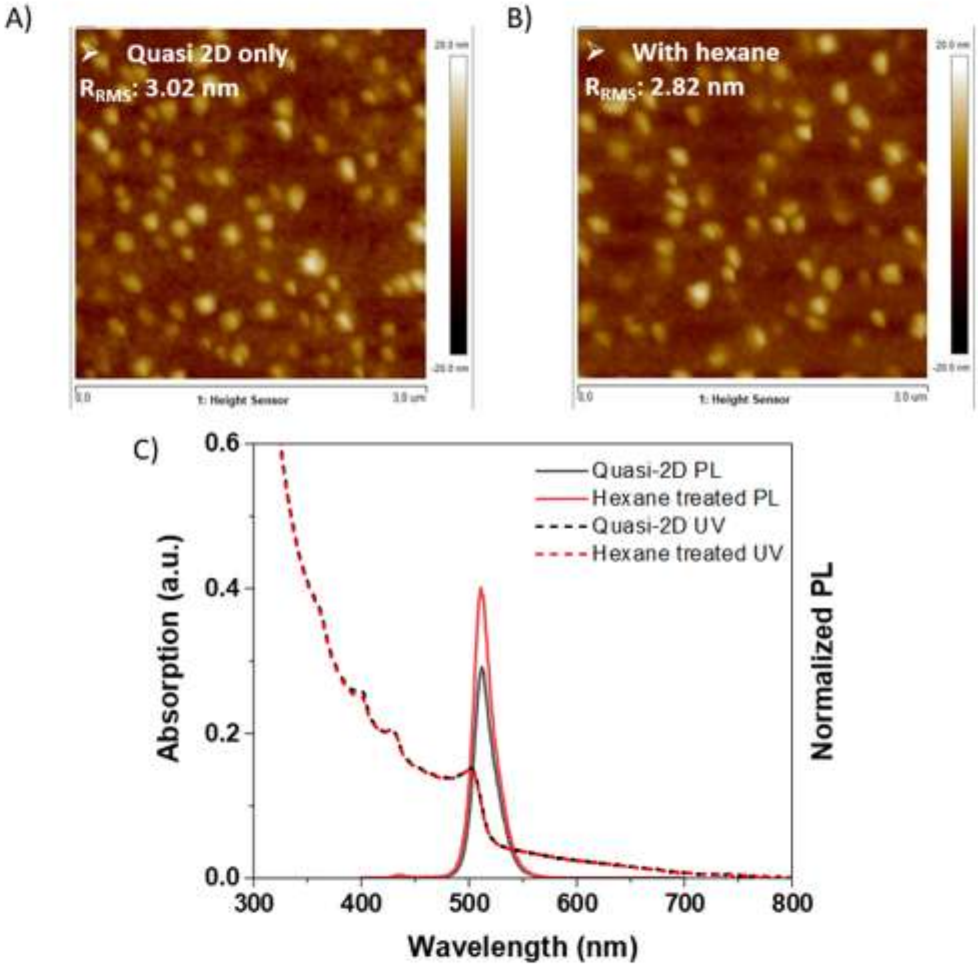

Figure S4. Atomic force microscopy images of a representative quasi-2D perovskite film acquired (a) before and (b) after the addition of hexane (10 μL). (c) Corresponding UV–vis absorption and PL emission spectra collected upon hexane addition.

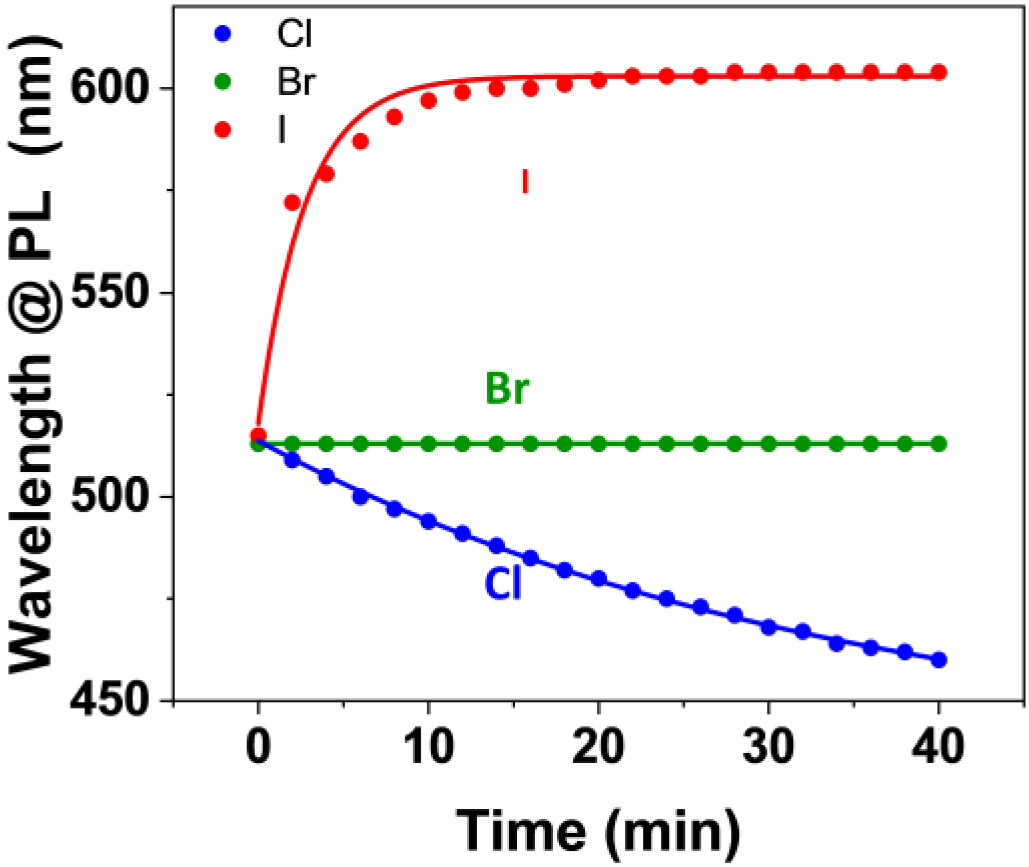

Figure S5. Evolution of band-edge PL emission wavelength with time after the CsX NC treatment. Note the shift of the PL emission wavelength to 570 nm immediately after the CsI NC addition.

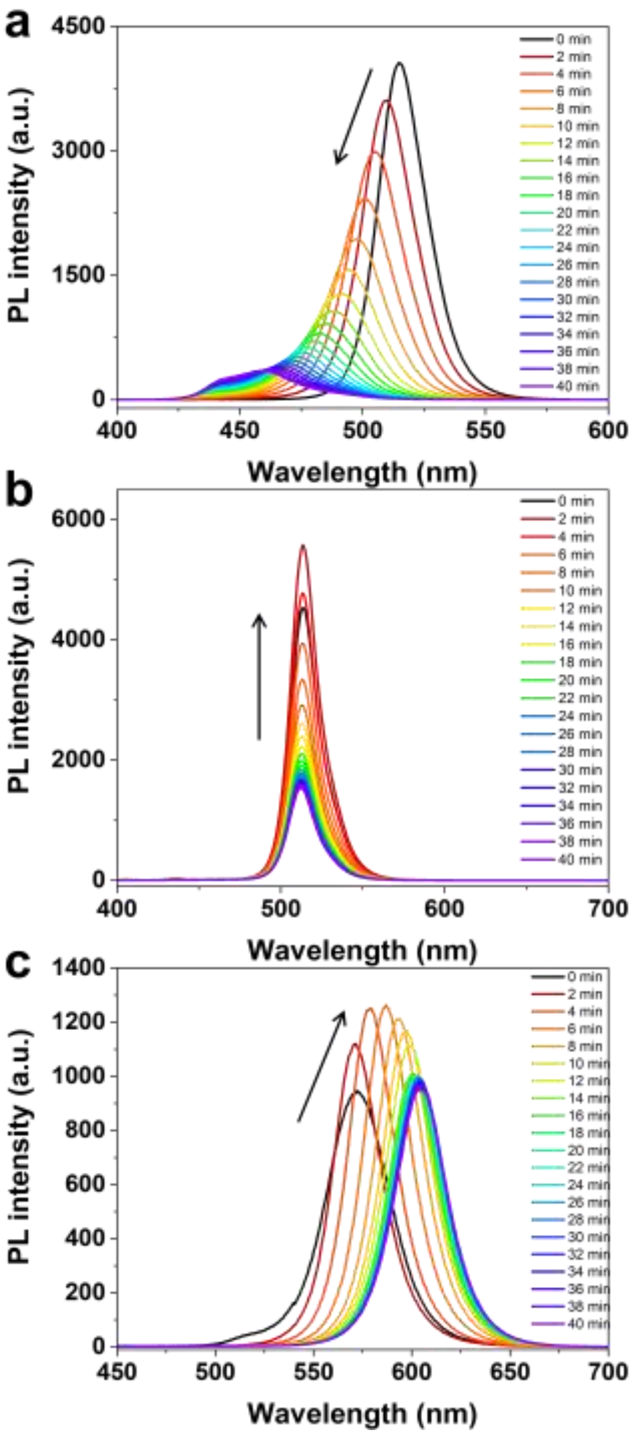

Figure S6. PL spectra recorded at room temperature for quasi-2D perovskite films treated with (a) CsCl, (b) CsBr, and (c) CsI NCs and corresponding to Figure 3(d), (e), and (f), respectively.

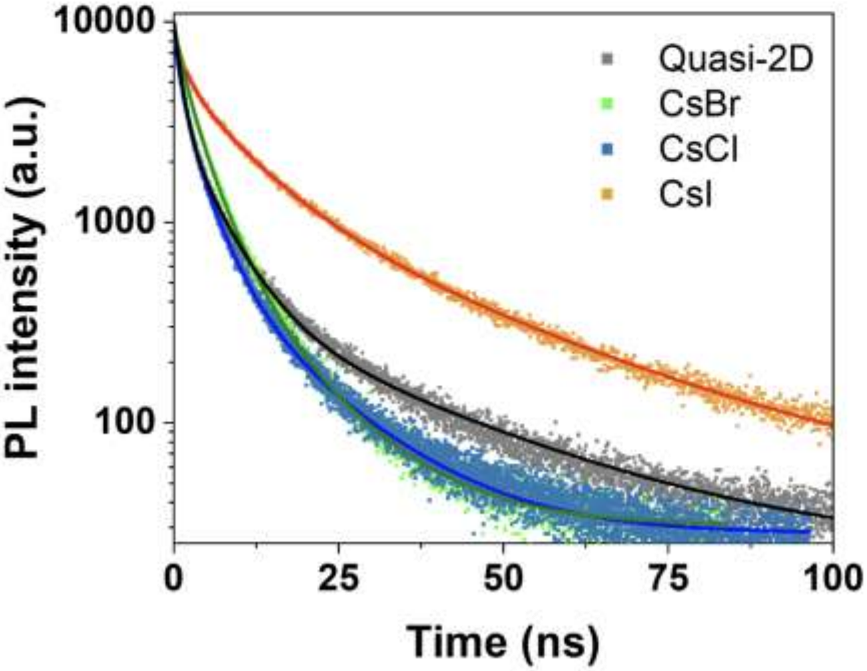

Figure S7. PL decay curves monitored using time-correlated single photon counting upon the addition of the CsX NCs and corresponding kinetic fitting parameters obtained using tri-exponential fitting for pristine quasi-2D perovskite (black) films and CsX NC-treated (Cl: blue, Br: green, and I: red) quasi-2D perovskite films, respectively

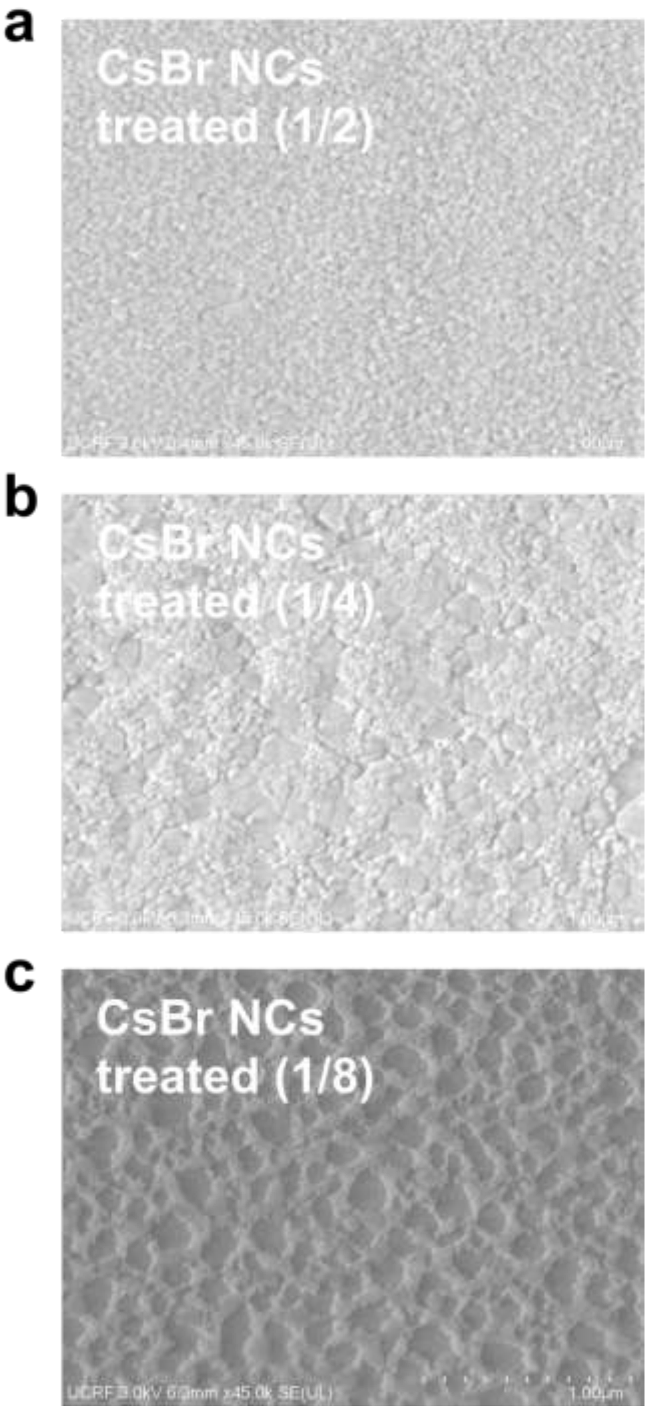

Figure S8. SEM images of quasi-2D perovskite films treated with a 10 μL of CsBr NC solutions (10 mg/ml), diluted with hexane at different ratios: (a) (NC solution):(hexane) = 1:1, (b) (NC solution):(hexane) = 1:3, and (c) (NC solution):(hexane) = 1:7.

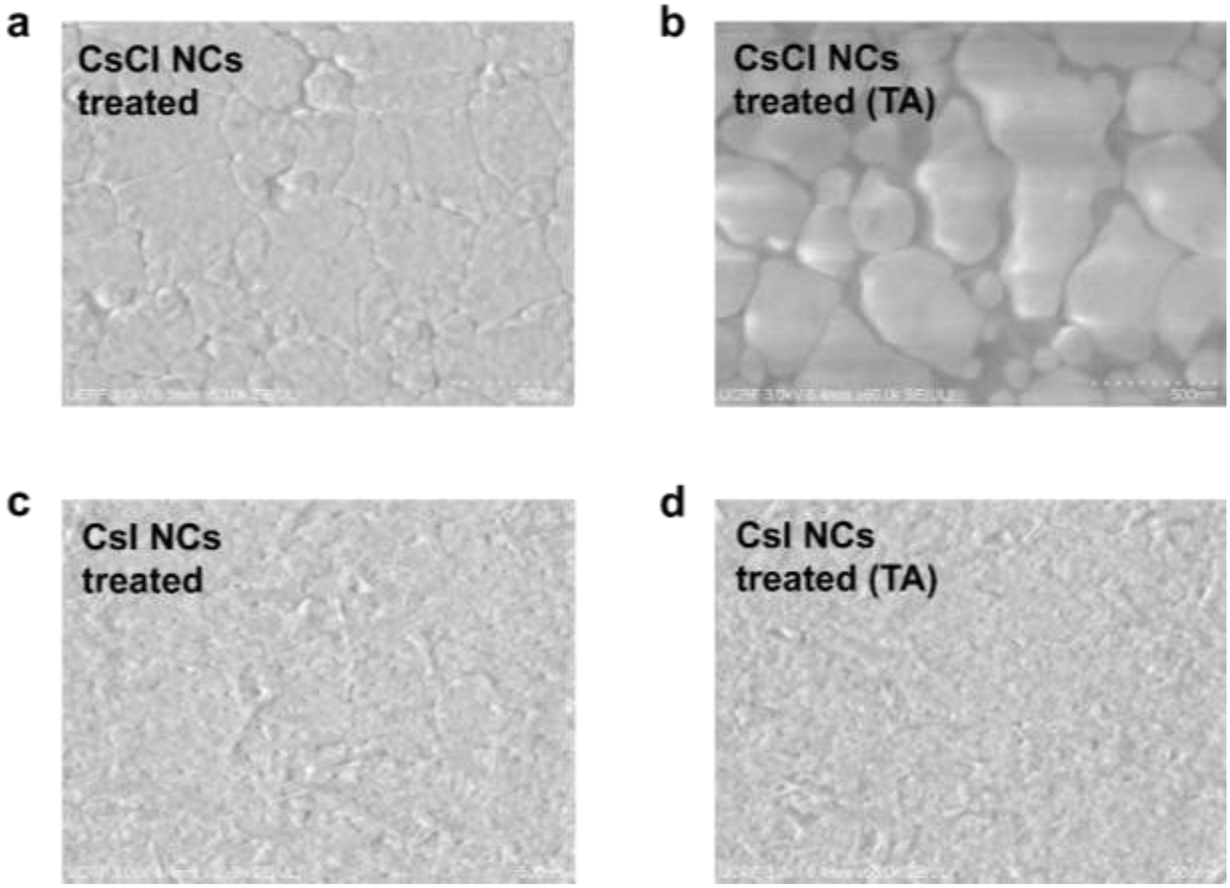

Figure S9. SEM images of quasi-2D perovskite films treated with the (a, b) CsCl and (c, d) CsI NCs (a, c) without and (b, d) with subsequent thermal annealing.

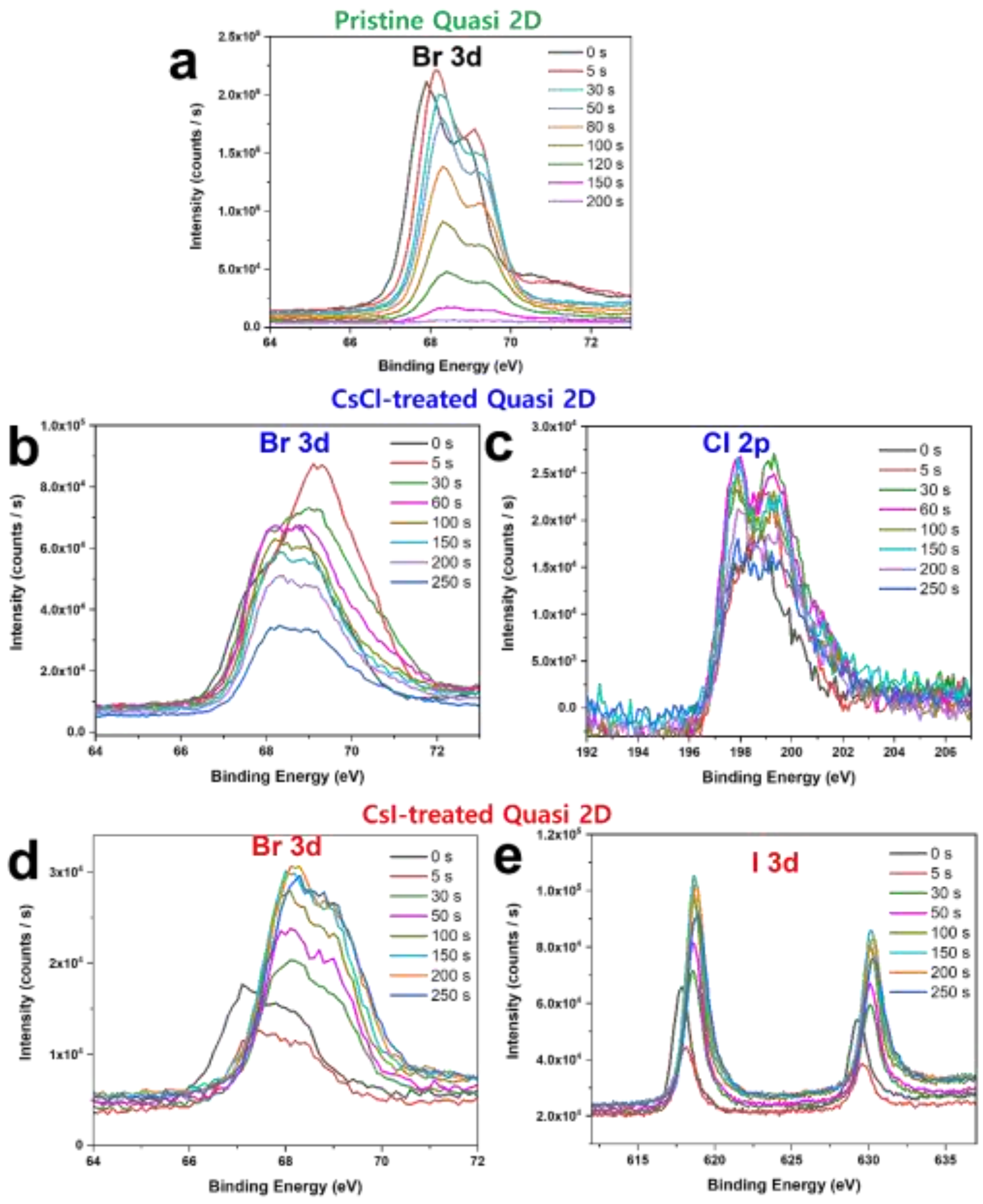

Figure S10. XPS spectra analysis for halides: (a) Br 3d spectra of pristine quasi-2D film, (b,c) Br 3d and Cl 2p of CsCl NC–treated quasi-2D films, and (d,e) Br 3d and I 3d of CsI NC–treated quasi-2D films, respectively.

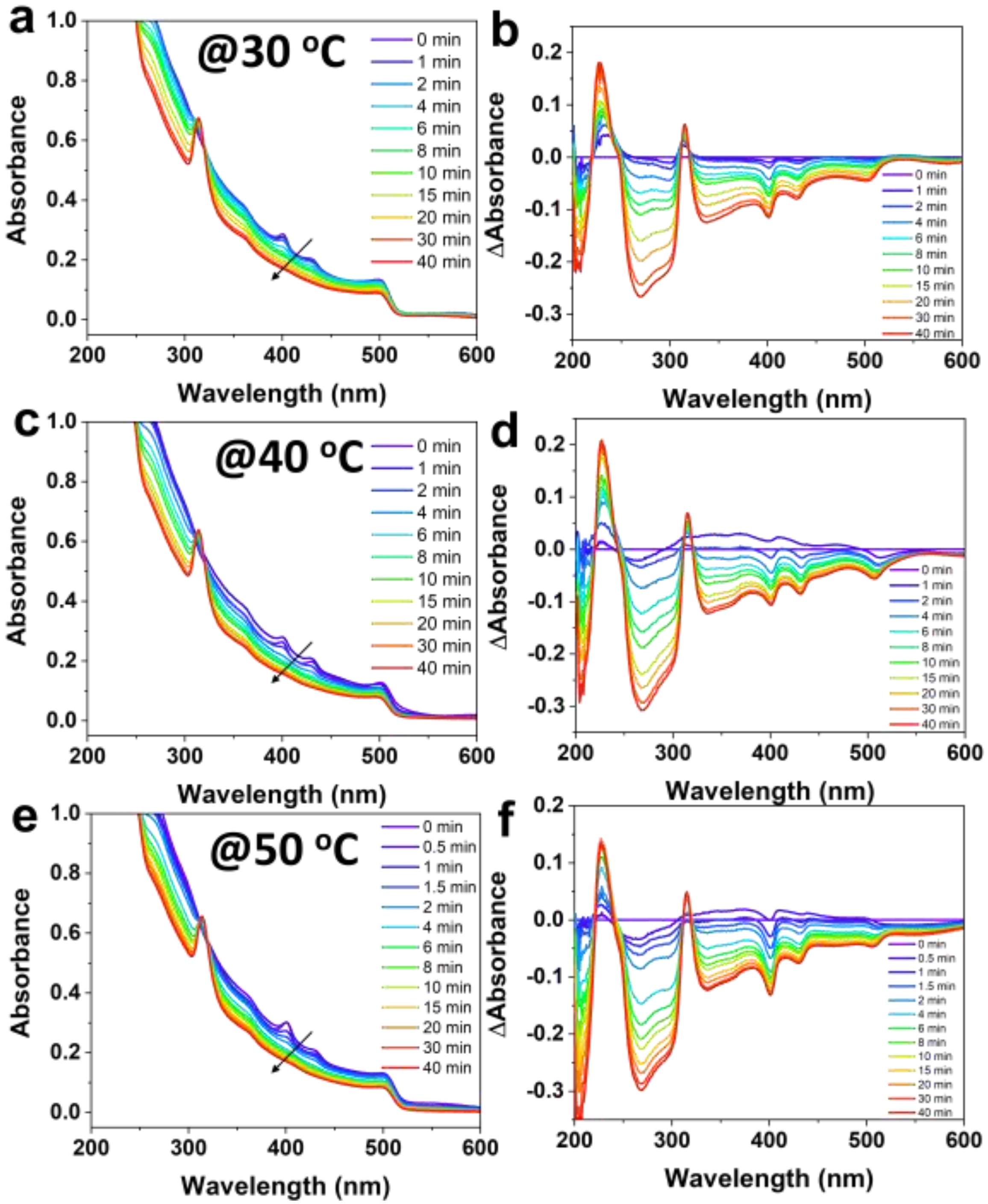

Figure S11. Time-dependent (a, c, e) absorption and (b, d, f) difference spectra recorded for CsCl NC–treated quasi-2D perovskite films on quartz at exchange reaction temperatures of (a, b) 30, (c, d) 40, and (e, f) 50 °C over 40 min. The absorption spectrum at 0 min served as a reference to obtain the difference spectra (Δ*A*).

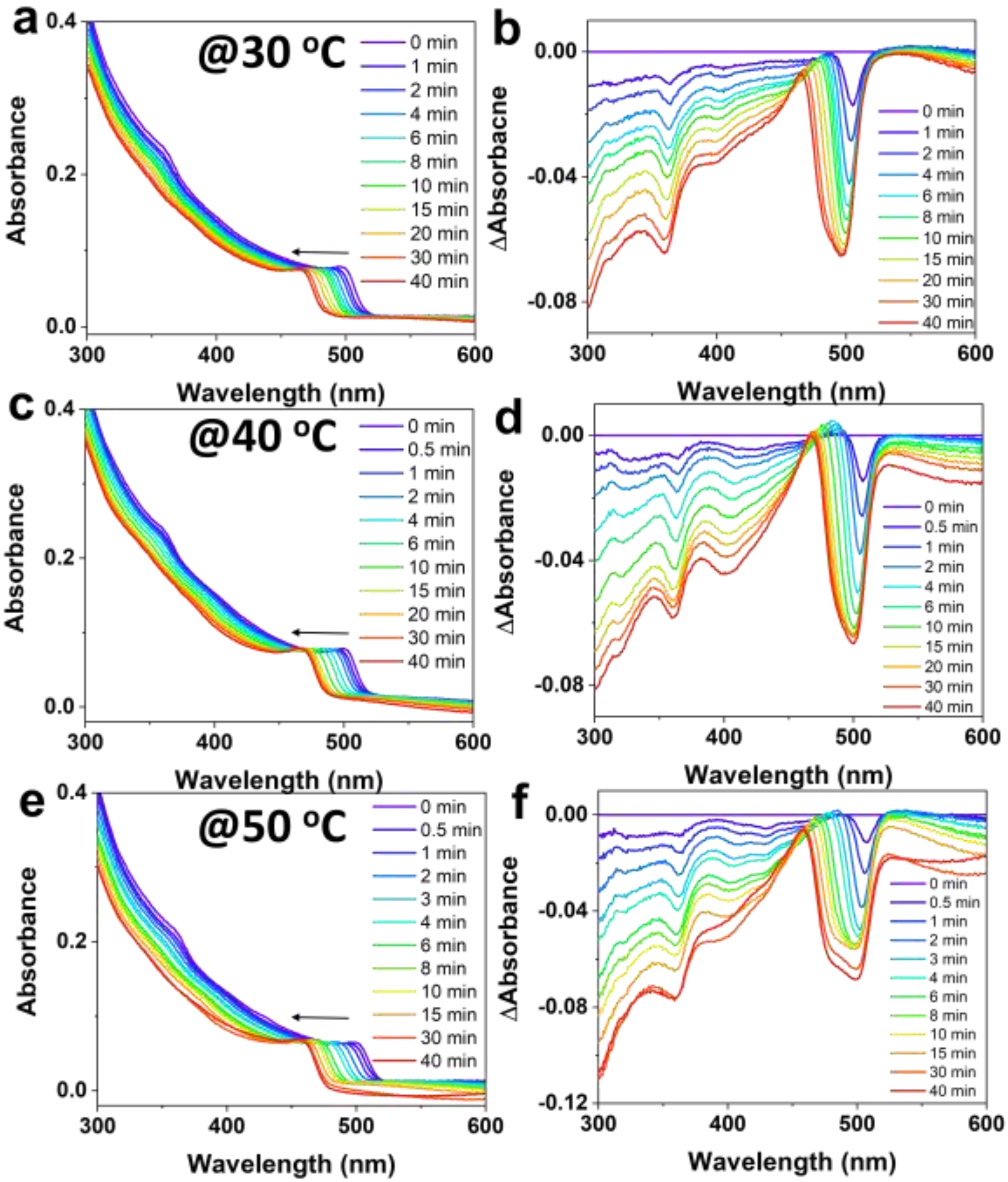

Figure S12. Time-dependent (a, c, e) absorption and (b, d, f) difference spectra recorded for CsCl NC–treated quasi-2D perovskite films on quartz at exchange reaction temperatures of (a, b) 30, (c, d) 40, and (e, f) 50 °C over 40 min. The absorption spectrum at 0 min served as a reference to obtain the difference spectra (ΔA).

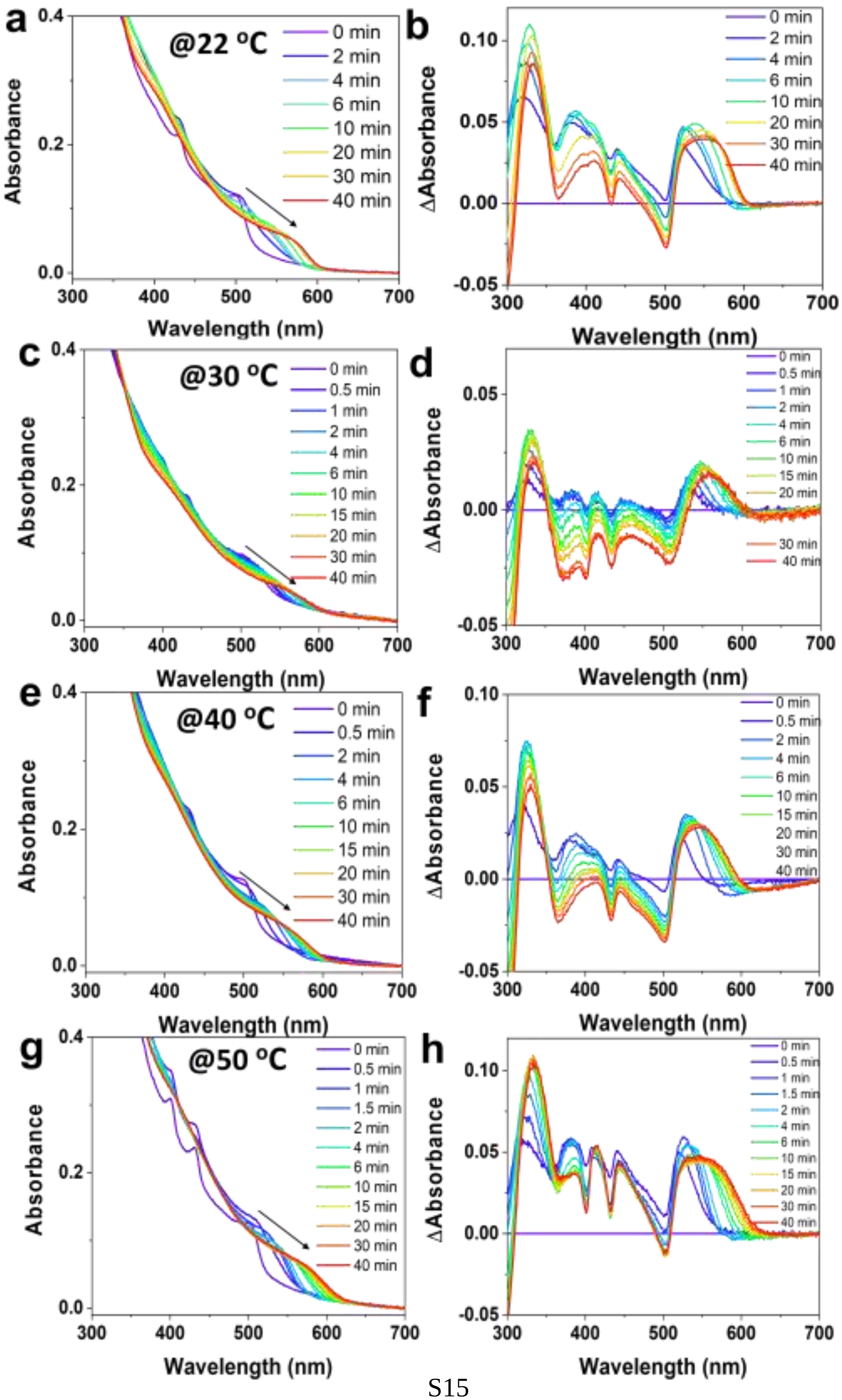
a
@22 °C
0 min
2 min
4 min
6 min
10 min
20 min
30 min
40 min
Absorbance
Wavelength (nm)
b
ΔAbsorbance
Wavelength (nm)
c
@30 °C
0 min
0.5 min
1 min
2 min
4 min
6 min
10 min
15 min
20 min
30 min
40 min
Absorbance
Wavelength (nm)
d
ΔAbsorbance
Wavelength (nm)
e
@40 °C
0 min
0.5 min
2 min
4 min
6 min
10 min
15 min
20 min
30 min
40 min
Absorbance
Wavelength (nm)
f
ΔAbsorbance
Wavelength (nm)
g
@50 °C
0 min
0.5 min
1 min
1.5 min
2 min
4 min
6 min
10 min
15 min
20 min
30 min
40 min
Absorbance
Wavelength (nm)
h
ΔAbsorbance
Wavelength (nm)

Figure S13. Time-dependent (a, c, e, g) absorption and (b, d, f, h) difference spectra recorded for CsI NC–treated quasi-2D perovskite films on quartz at exchange reaction temperatures of (a, b) 22, (c, d) 30, (e, f) 40, and (g, h) 50 °C over 40 min. The absorption spectrum at 0 min served as a reference to obtain the difference spectra (ΔA).

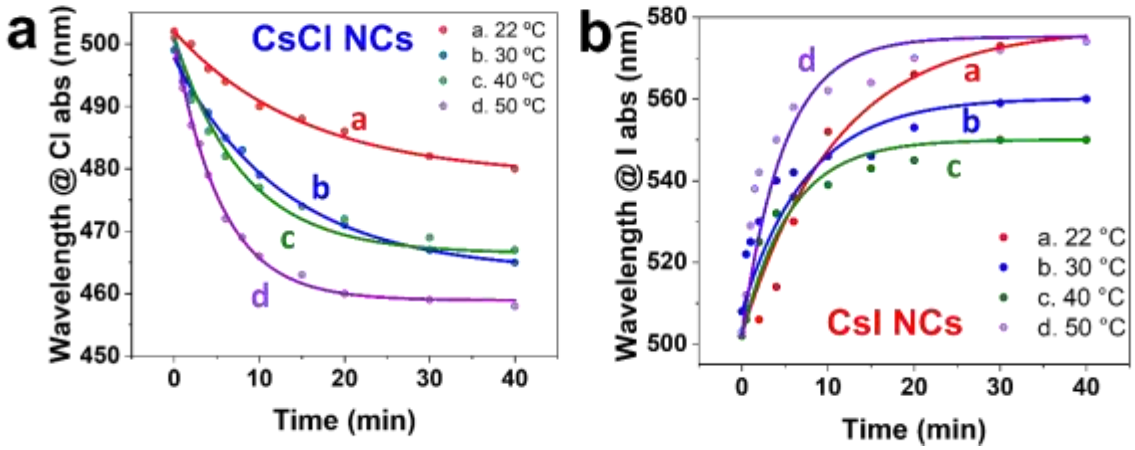

Figure S14. Kinetic traces and their monoexponential fits obtained for quasi-2D perovskite films treated with the (a) CsCl and (b) CsI NCs at 22–50 °C. Note that monoexponential fittings were used for the determination of rate constant (k).

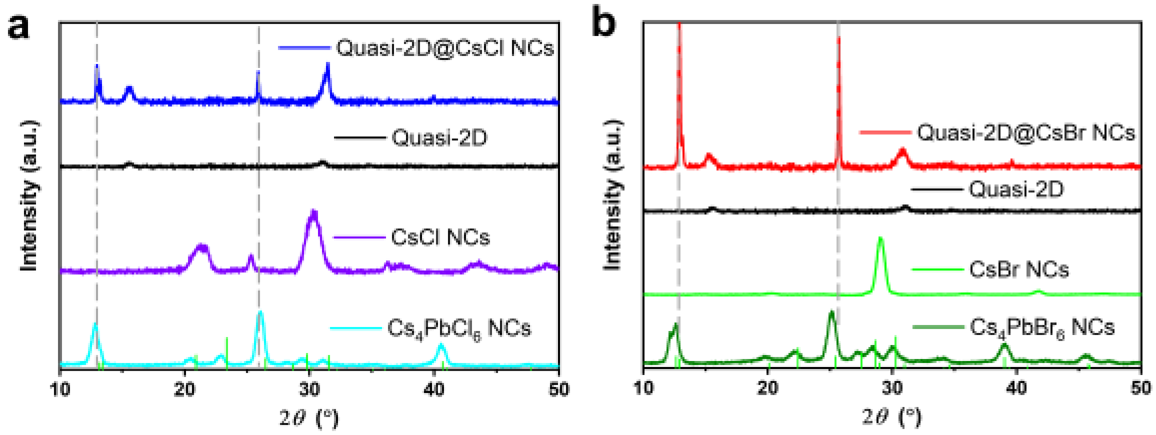

Figure S15. X-ray diffraction patterns of (a) CsCl and (b) CsBr NC–treated quasi-2D perovskite films recorded 12 h after treatment.

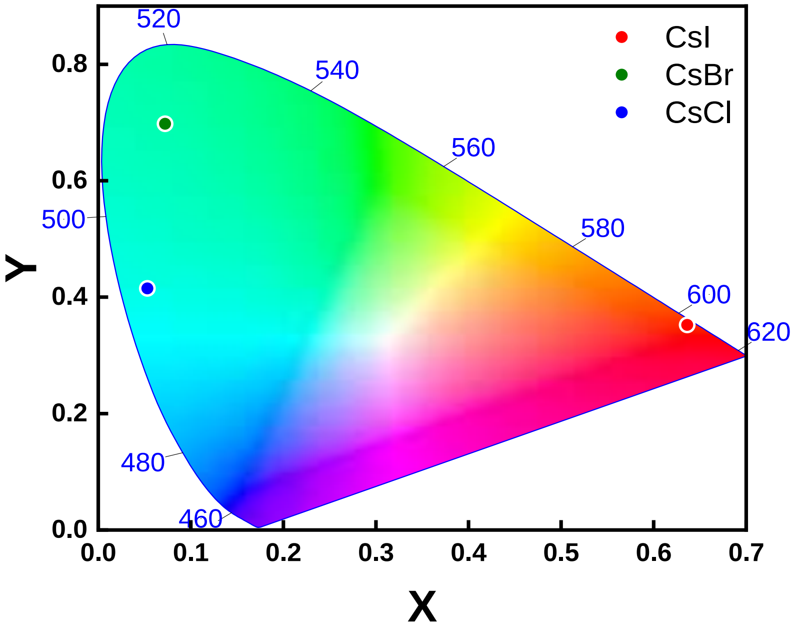

Figure S16. CIE coordinates of perovskite light-emitting diodes (PeLEDs) after treatment with the CsX NCs (2.5 mg mL^{-1}).

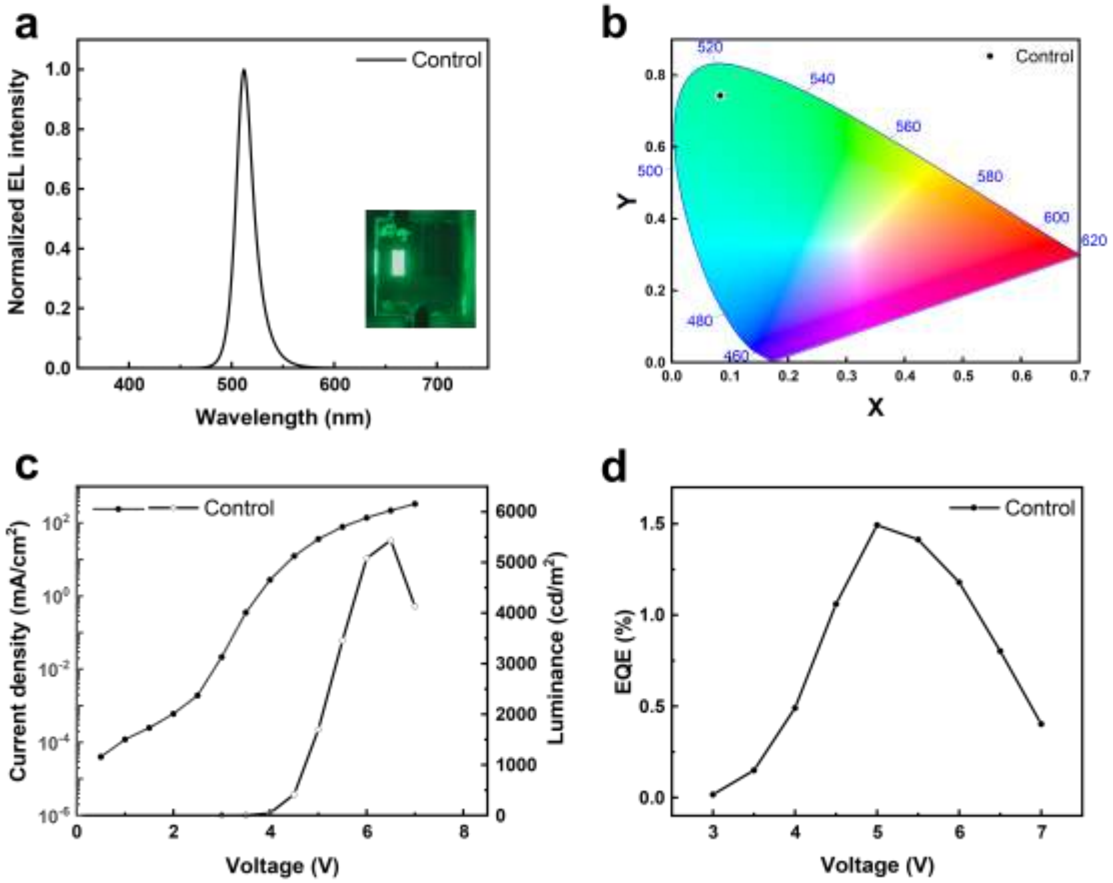

Figure S17. (a) EL spectrum, (b) CIE coordinates, (c) *J*–*V*–*L* plot, and (d) EQE–*V* plot of the control (CsX NC–untreated) PeLED.

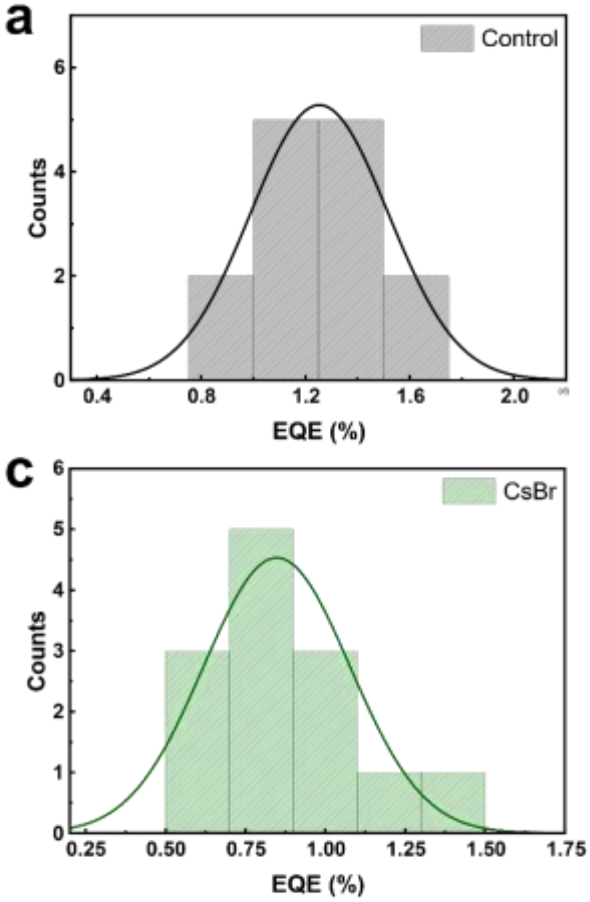

Figure S18. Performance reproducibilities of (a) the control device and (b) CsI-, (c) CsBr-, and (d) CsCl-treated PeLEDs.

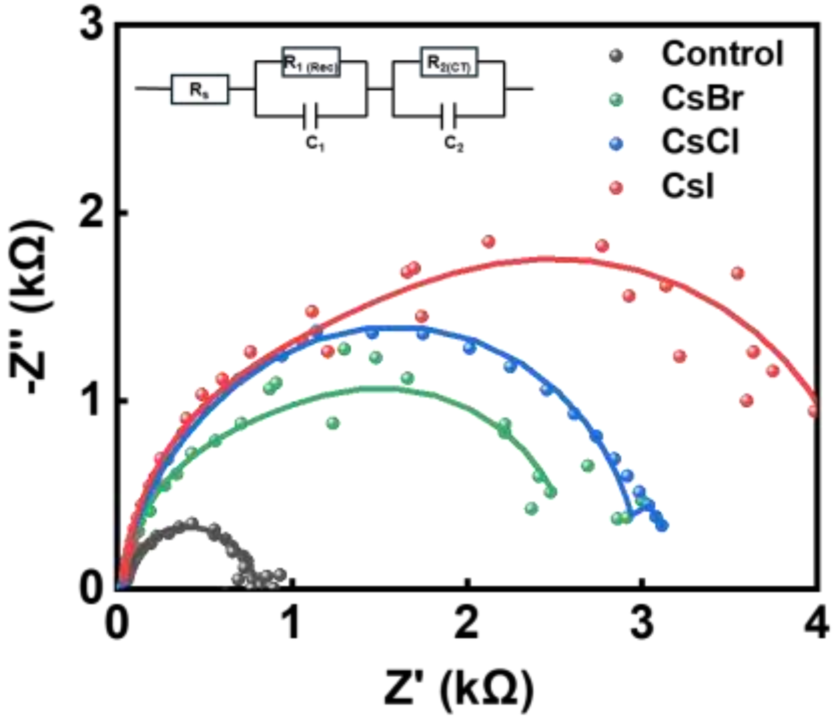

Figure S19. EIS spectra of the PeLEDs of the control and CsX-treated devices. The inset shows the equivalent circuit model.

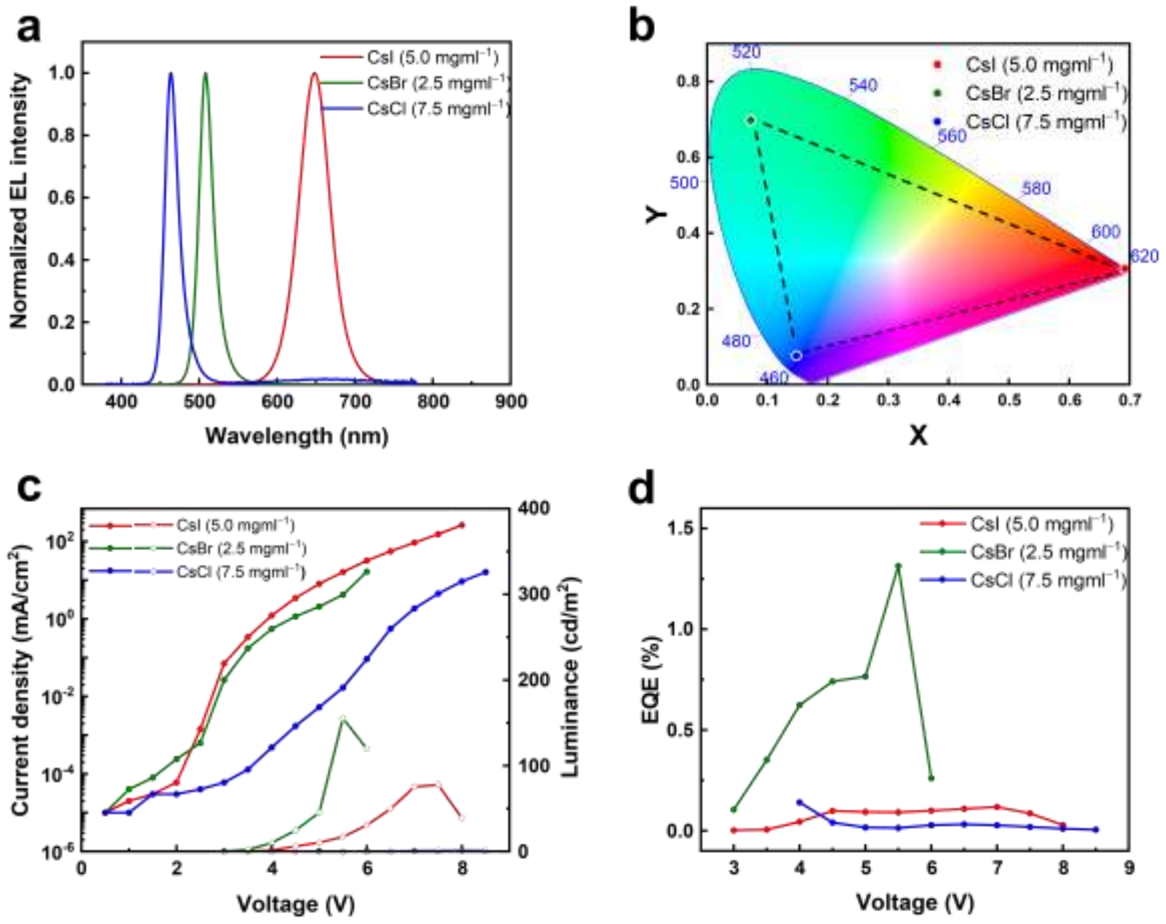

Figure S20. Characteristics of PeLEDs based on CsX NC–treated quasi-2D perovskite films. (a) Electroluminescence (EL) spectra, (b) CIE coordinates, (c) *J*–*V*–*L* plots, and (d) external quantum efficiency (EQE)–voltage (*V*) plots. CsI, CsBr, and CsCl concentrations of 5.0, 2.5, and 7.5 mg mL^{-1}, respectively, were used.

	A_1	τ_1 (ns)	A_2	τ_2 (ns)	A_3	τ_3 (ns)	τ_{avg} (ns)
**Quasi-2D**	**0.66**	**1.13**	**0.30**	**5.38**	**0.04**	**28.05**	**11.86**
**Quasi-2D@ CsBr**	**0.37**	**1.50**	**0.55**	**4.08**	**0.08**	**11.76**	**5.69**
**Quasi-2D@ CsCl**	**0.49**	**1.07**	**0.44**	**3.69**	**0.08**	**13.55**	**6.44**
**Quasi-2D@ CsI**	**0.36**	**1.93**	**0.47**	**9.21**	**0.17**	**31.23**	**20.14**

Table S1. PL lifetimes of pristine and CsX NC–treated quasi-2D perovskite films determined by triexponential fitting.

	L_{max} [cdm^{-2}] @ bias [V]	LE_{max} [cdA^{-1}] @ bias [V]	EQE_{max} [%] @ bias [V]	EL [nm]	Turn-on Voltage [V] @ 0.1 cd/m^{-2}
Control	**5438.5 @ 6.5**	**4.655 @ 5.0**	**1.492 @ 5.0**	**512**	**3.5**
CsI	**184.57 @ 7.5**	**0.207 @ 7.0**	**0.157 @ 7.0**	**637**	**4.0**
CsBr	**155.38 @ 5.5**	**3.682 @ 5.5**	**1.314 @ 5.5**	**508**	**3.5**
CsCl	**139.6 @ 8.5**	**2.058 @ 5.0**	**1.235 @ 5.0**	**494**	**4.5**

Table S2. Characteristics of control and CsX-treated PeLEDs.
